# The role of pioneering transcription factors, chromatin accessibility and epigenetic reprogramming in oncogenic viruses

**DOI:** 10.3389/fmicb.2025.1602497

**Published:** 2025-06-16

**Authors:** Mankgopo Kgatle, Saidon Mbambara, Leon Khoza, Olalekan Fadebi, Tivani Mashamba-Thompson, Mike Sathekge

**Affiliations:** ^1^Department of Nuclear Medicine, University of Pretoria and Steve Biko Academic Hospital, Pretoria, South Africa; ^2^Nuclear Medicine Research Infrastructure (NuMeRI), Steve Biko Academic Hospital, Pretoria, South Africa; ^3^Department of Medicine, University of Cape Town and Groote Schuur Hospital, Cape Town, South Africa; ^4^Department of Biomedical Sciences, Tropical Diseases Research Center, Ndola, Zambia; ^5^Faculty of Health Sciences, School of Health System and Public Health, University of Pretoria, Pretoria, South Africa

**Keywords:** chromatin accessibility, DNA methylation, EBV, epigenetics, epigenetics reprogramming, oncogenic viruses, pioneer transcription factors

## Abstract

Oncogenic viruses typically manipulate host cellular mechanisms to drive tumorigenesis. They exploit pioneering transcription factors to modify gene expression, enabling uncontrolled proliferation. These viruses alter chromatin accessibility and induce chromatin remodelling, disrupting DNA repair and promoting viral genome integration. Additionally, epigenetic reprogramming through mechanisms like DNA methylation and histone modifications silences tumor suppressor genes and activates oncogenes. Understanding these mechanisms is critical for identifying more improved therapeutic targets, improving diagnostics, and predicting disease progression. Advances in this field can guide the development of innovative treatments and early detection tools. This comprehensive review synthesizes existing knowledge on the contributions of oncogenic viruses such as hepatitis B virus (HBV), hepatitis C virus (HCV), human papillomavirus (HPV), and human T-cell leukaemia virus type 1 (HTLV-1), Epstein–Barr virus (EBV), human herpesvirus 8 (HHV-8), and Merkel cell polyomavirus (MCV) to cancer development, highlighting their therapeutic relevance and driving forward research in viral oncogenesis.

## Introduction

Oncogenic viruses such as hepatitis B virus (HBV), hepatitis C virus (HCV), human papillomavirus (HPV), human T-cell leukaemia virus type 1 (HTLV-1), Epstein–Barr virus (EBV), human herpesvirus 8 (HHV-8), and Merkel cell polyomavirus (MCV) significantly influence host cellular mechanisms to drive oncogenesis (Mohanty and Harhaj, 2023; Krump and You, 2018). These viruses significantly influence host cellular mechanisms by disrupting transcription factor activity, altering chromatin accessibility, and reprogramming epigenetic processes (Krump and You, 2018; Cirillo et al., 2002). Such disruptions lie at the heart of their role in tumorigenesis (McLaughlin-Drubin and Münger, 2009; Seeger and Mason, 2000; Young and Rickinson, 2004; Mesri et al., 2014).

Many oncogenic viruses integrate into accessible regions of the host genome, activating oncogenes while silencing tumor suppressor genes (McLaughlin-Drubin and Münger, 2009; Seeger and Mason, 2000; Young and Rickinson, 2004; Mesri et al., 2014). This process disrupts normal transcriptional activities to establish persistent infections, promote cell survival, and fuel oncogenesis (Young and Rickinson, 2004; Mesri et al., 2014). Particularly, pioneer transcription factors (PTFs) are often co-opted by these viruses to remodel condensed chromatin and recruit other proteins, thereby enhancing access to the transcriptional machinery essential for gene expression (Cirillo et al., 2002). The reprogramming facilitated by these PTFs involves key epigenetic modifications such as chromatin remodelling, histone alterations, and DNA methylation (Iwafuchi-Doi and Zaret, 2014). Oncogenic viruses, including HBV, HCV, HPV, HTLV-1, EBV, HHV-8, and MCV utilize these mechanisms to manipulate host chromatin, thereby advancing disease progression (Krump and You, 2018).

Chromatin accessibility further determines which genomic regions are available for transcription (Clapier et al., 2017). Oncogenic viruses like HBV and HPV, which integrate into the host genome, can exploit local chromatin states to modulate host gene expression. Although EBV typically persists as an episome, it can still impact host chromatin structure and gene regulation by interacting with epigenetic modifiers, even without integration. However, in certain cases, such as certain lymphomas and EBV-associated gastric carcinomas, EBV may integrate into the host genome (Péneau et al., 2022; Dai et al., 2021). This chromatin remodelling activity interferes with DNA repair mechanisms and apoptosis, ultimately fostering genomic instability and supporting viral genome integration into the host DNA—processes that collectively drive tumorigenesis (Dai et al., 2021).

Epigenetic reprogramming is another hallmark of viral oncogenesis (Krump and You, 2018). Oncogenic viruses deploy mechanisms like DNA methylation and histone modification to deactivate tumor suppressor genes and activate oncogenes (Warburton et al., 2021). For example, EBV and HTLV-1 leverage viral proteins to manipulate the host’s epigenetic machinery, thereby creating a cellular environment conducive to cancer development (Dai et al., 2021; Banerjee et al., 2024; Soliman et al., 2021). These changes are essential for maintaining viral survival while promoting tumor progression (Soliman et al., 2021).

A deeper understanding of these processes is critical for advancing cancer diagnostics, identifying therapeutic targets, and developing strategies to impede disease progression. This review consolidates current knowledge on the roles of transcription factors, chromatin remodelling, and epigenetic reprogramming in viral-induced malignancies, emphasizing their importance in therapeutic research and clinical applications. The focus of our study on HBV, HCV, HPV, HTLV-1, EBV, HHV8, and MCV stems from their classification as oncogenic viruses due to their direct role in causing cancers. By disrupting cellular mechanisms through pathways such as transcription factor activity, chromatin accessibility, and epigenetic reprogramming, these viruses remain central to the scope of our study.

## A “peek” into PTFS and epigenetics

### Pioneer transcription factors and chromatin remodelling

PTFs are a unique class of transcription factors that can bind to closed, heterochromatic regions and initiate chromatin remodelling (King and Klose, 2017; Soufi et al., 2015; Sinha et al., 2023). They are termed “pioneer factors” because they can “open” or remodel chromatin at specific genomic sites, making these regions transcriptionally active. Unlike conventional transcription factors, PTFs interact with nucleosome-bound DNA, thus unlocking previously inaccessible DNA sequences and make them transcriptionally active (Cirillo et al., 2002; Soufi et al., 2015). By recruiting chromatin remodelers, histone modifiers, and DNA methylation machinery, PTFs establish active or poised transcriptional states that drive gene transcription (Soufi et al., 2015; Sinha et al., 2023). These factors are critical in oncogenic virus-mediated transformation, as they reprogram host chromatin landscapes to activate both viral and host oncogenes while silencing tumor suppressor genes (Neugebauer et al., 2023).

### Key mechanisms of epigenetic regulation

Epigenetic regulation controls gene expression without altering the underlying DNA sequence (Cavalli and Heard, 2019). This process involves mechanisms such as chromatin remodelling, histone modifications, and DNA methylation, which collectively influence chromatin structure and gene accessibility (Jaenisch and Bird, 2003). Chromatin, composed of DNA and histone proteins, exists in two main states: euchromatin, which is loosely packed and transcriptionally active, and heterochromatin, which is tightly packed and repressive (Lippman et al., 2004; Morrison and Thakur, 2021; Teperino et al., 2010; Zhang et al., 2015). ATP-dependent complexes such as chromodomain helicase DNA-binding (CHD), switch/sucrose non-fermentable (SWI/SNF), imitation switch (ISWI), and INO80, dynamically reorganize chromatin to regulate gene accessibility (Clapier et al., 2017). Proper chromatin is essential for biological processes like stem cell differentiation, stress responses, and cancer progression, while disruptions in these processes can lead to abnormal gene expression and tumorigenesis (Clapier et al., 2017; Mansisidor and Risca, 2022).

**Figure 1:**
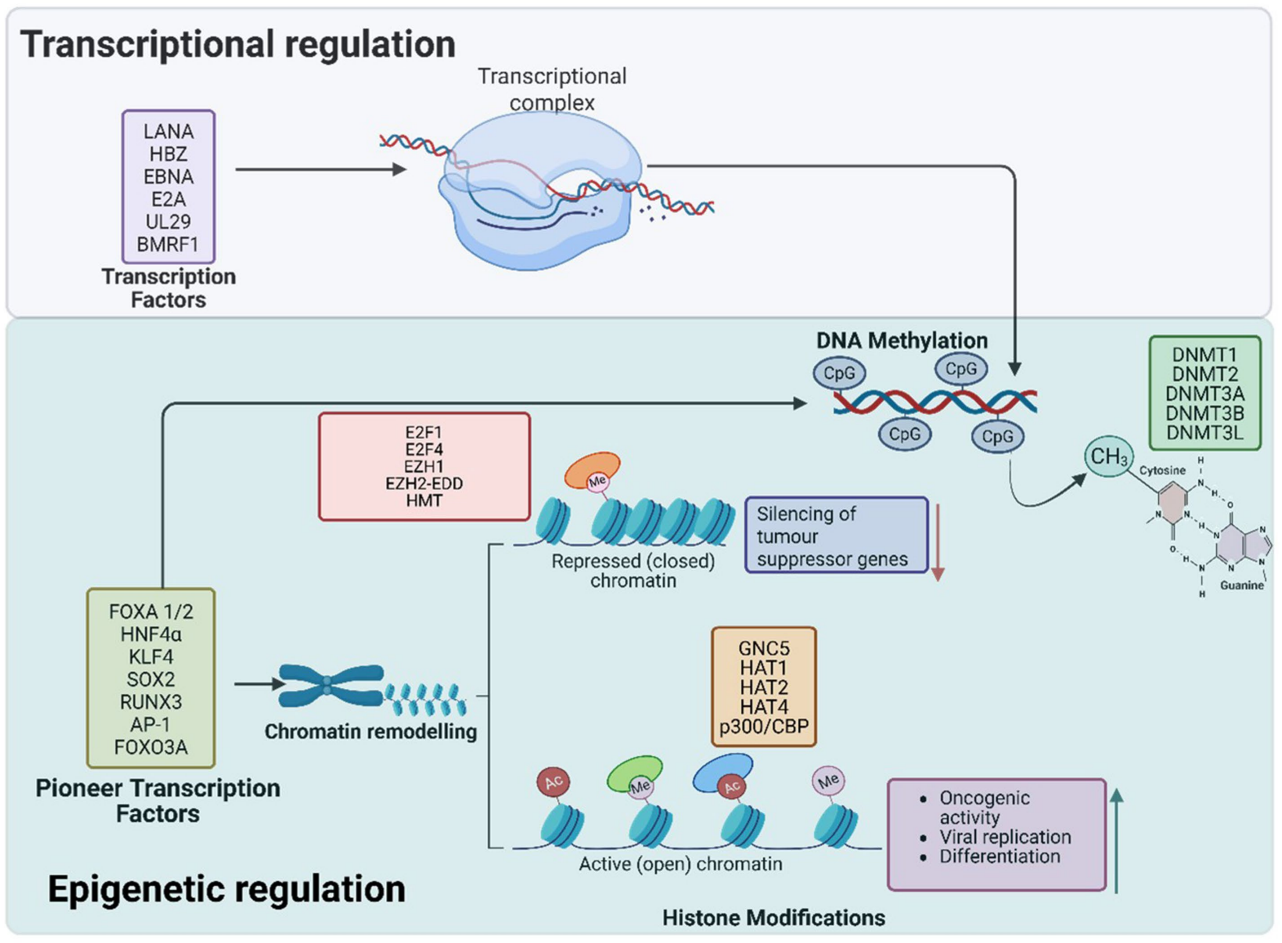
Pioneer transcription factors play a critical role in chromatin remodelling and epigenetic reprogramming during oncogenic transformation. They bind to closed or opened chromatin regions, facilitating the recruitment of chromatin remodelers and histone-modifying enzymes to establish active or repressive transcriptional states. Through histone acetylation and methylation, PTFs activate oncogenes such as *TERT* and *CCNE1* while repressing tumor suppressor genes like *p16*, *pRB*, and *PTEN*. Additionally, DNA methylation at CpG sites further reinforces the silencing of tumor suppressor genes, promoting immune evasion and cellular transformation. Collectively, these epigenetic modifications disrupt normal cellular function, driving cancer progression. The transcription factors in the upper purple box are viral in origin.

Histone proteins, which form the core of nucleosomes, undergo post-translational modifications (PTMs) that regulate chromatin structure and gene expression (Pietropaolo et al., 2021). These modifications include acetylation, methylation, phosphorylation, ubiquitination, and SUMOylation (Jones and Baylin, 2007). For instance, acetylation by histone acetyltransferases (HATs) loosens histone-DNA interactions to promote gene transcription, while histone methylation can either activate or repress transcription, depending on the specific marker (Mac and Moody, 2020). Aberrant histone modifications are often associated with cancer, as they disrupt genes vital for tumor suppression and immune responses (Figure 1).

### DNA methylation and demethylation

DNA methylation, the addition of methyl groups to cytosine residues at CpG dinucleotides, is typically associated with transcriptional repression (Moore et al., 2013). This process is catalyzed by DNA methyltransferases (DNMTs), including DNMT1, DNMT2, and DNMT3. Hypermethylation in promoter regions silences tumor suppressor genes, whereas hypomethylation activates oncogenes, promoting uncontrolled cell proliferation (Andreescu, 2024; Varley et al., 2013). Global DNA hypomethylation, common in aging and cancer, can also lead to genomic instability and activation of transposable elements (Varley et al., 2013). Therapeutically, DNMT inhibitors like azacitidine and decitabine are used therapeutically to reactivate silenced tumor suppressor genes.

Conversely, DNA demethylation is mediated by ten-eleven translocation (TET) enzymes—TET1, TET2, and TET3—which convert 5-methylcytosine to intermediate products that restore cytosine to its unmethylated state. TET enzymes are essential for embryonic development and stem cell differentiation (Ito et al., 2010; Ono et al., 2021; Tahiliani et al., 2009). Reduced TET activity can result in hypermethylation of tumor suppressor genes, contributing to cancer development (Thienpont et al., 2016; Rasmussen and Helin, 2016).

### Histone methylation and polycomb repressive complexes

Histone methylation, orchestrated by histone methyltransferases (HMTs) and demethylases (HDMs), plays a key role in transcriptional regulation (Klose and Zhang, 2007). Certain methylation marks, such as H3K4me3 and H3K36me3, are linked to transcriptional activation, while others, such as H3K27me3 and H4K20me3, are associated with transcriptional repression (Ratner, 2021). The repressive mark H3K27me3, catalyzed by the polycomb repressive complex 2 (PRC2) via its Enhancer of Zeste Homolog 1 / 2 (EZH2/EZH1) subunits, is a critical signal for PRC activity. This mark recruits PRC1, which ubiquitinates histone H2A at lysine 119 (H2AK119Ub), leading to chromatin compaction and gene silencing (Martin and Moorehead, 2020). H3K27me3 is vital for repressing developmental genes, such as HOX genes, and for maintaining cell cycle regulation. Loss of this mark can result in differentiation defects, loss of stem cell identity, and cancer progression (Jones and Baylin, 2007).

Additionally, certain histone marks can counteract PRC-mediated repression. For example, genes with both H3K4me3 (an active mark) and H3K27me3 exist in a “bivalent” state in stem cells, allowing them to remain poised for either activation or repression. Other marks, such as H3K9me3, generally do not overlap with PRC activity, illustrating distinct silencing pathways. Additional histone modifications, such as phosphorylation, ubiquitination, and SUMOylation, influence chromatin dynamics, DNA repair, and gene expression (Ryu and Hochstrasser, 2021).

## Hepatitis B virus and hepatocellular carcinoma

### HBV virology

HBV is a partially double-stranded DNA virus belonging to the *Hepadnaviridae* family that primarily targets hepatocytes, causing hepatitis B (Krump and You, 2018; Karayiannis, 2017) It is recognized as a major oncogenic virus, contributing to hepatocellular carcinoma (HCC) through persistent infection, chronic inflammation, and epigenetic reprogramming (Karayiannis, 2017; Kgatle et al., 2017). HBV’s small, circular DNA genome (~3.2 kb) contains four overlapping open reading frames (ORFs) encoding essential viral proteins: the S gene (surface antigen, HBsAg), the C gene (core antigen, HBcAg, and HBeAg), the P gene (DNA polymerase), and the X gene (encoding HBx protein, which plays a key role in viral replication and oncogenesis) (Karayiannis, 2017; Robinson et al., 1974).

Unlike most DNA viruses, HBV replicates through an RNA intermediate, utilizing reverse transcription. Upon infection, the viral genome is converted into covalently closed circular DNA (cccDNA) in the nucleus, which serves as a template for transcription (Karayiannis, 2017). HBV is transmitted through blood, sexual contact, and perinatal routes from mother to child (di Filippo Villa and Navas, 2023).

Clinically, HBV infection can range from asymptomatic to acute hepatitis, presenting with symptoms such as jaundice, fatigue, and liver inflammation (Mistry and Yeoman, 2023). Chronic HBV infection significantly increases the risk of HCC development due to its persistent impact on host cellular and molecular mechanisms.

HBV integrates into the host genome, leading to long-term disruptions in chromatin accessibility and gene regulation (Péneau et al., 2022). HBV DNA integrates into active chromatin regions, where it modifies histone and DNA methylation marks to alter host gene expression (Péneau et al., 2022). These changes activate oncogenes and silence tumor suppressor genes, contributing to oncogenesis (Karayiannis, 2017; Kgatle et al., 2017). This capacity to regulate host epigenetics is central to HBV’s role in promoting hepatocyte transformation and the development of HCC (Péneau et al., 2022; Kgatle et al., 2017).

## HBV-mediated regulation of PTFS

**Figure 2:**
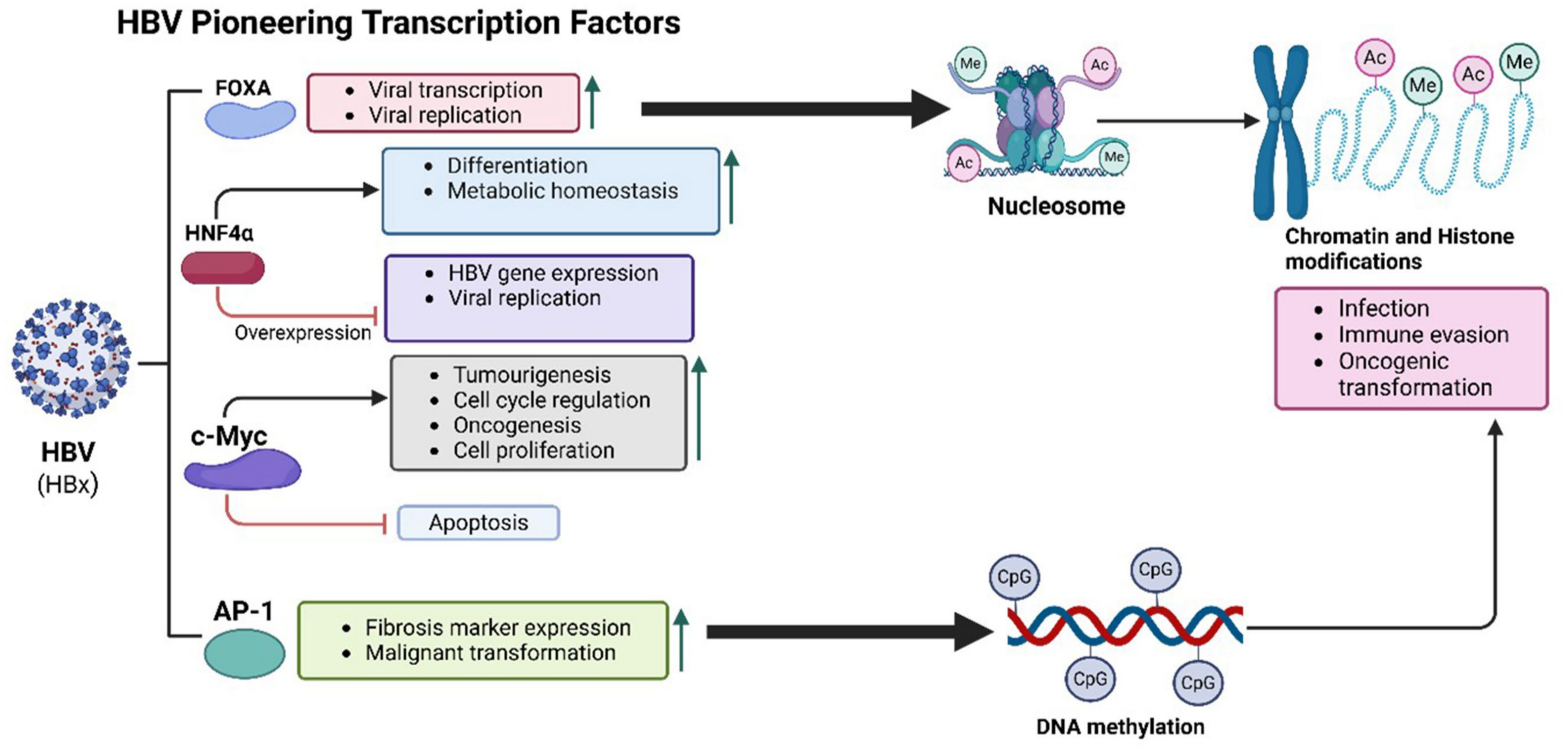
HBV-mediated PTFs regulation, chromatin remodelling and epigenetic reprogramming, and role in oncogenesis. The HBx protein of HBV plays a central role in oncogenesis by recruiting histone-modifying enzymes to alter chromatin structure, enhancing histone acetylation and methylation. These modifications activate oncogenes and silence tumor suppressor genes. HBV integrates into actively transcribed chromatin regions, such as *TERT* and *CCNE1* loci, disrupting cell cycle regulation and promoting tumor development. Interactions between HBx and transcription factors like FOXA1 and FOXA2 further increase chromatin accessibility and transcriptional activity, activating pro-oncogenic pathways and enabling immune evasion. Additionally, DNA methylation at CpG islands silences tumor suppressor genes, driving chronic liver disease and hepatocyte transformation.

HBV utilizes PTFs to remodel chromatin, enhancing oncogenic pathway activation, sustaining viral replication, and altering hepatocyte identity as described in Figure 2. These changes contribute to chronic liver disease and the progression to hepatocellular carcinoma (HCC) (Levrero and Zucman-Rossi, 2016).

Key liver-specific *PTFs* involved in HBV-mediated oncogenesis include the Forkhead box A (FoxA) family, particularly FoxA1 and FoxA2 (Chen et al., 2024). These transcription factors are essential for hepatic development and function, facilitating transcription by opening chromatin at liver-specific gene loci (Lee et al., 2005). HBV’s regulatory protein HBx interacts directly with FOXA1 and FOXA2, enhancing their chromatin-binding ability and transcriptional activity (Liu et al., 2023). This interaction activates pro-oncogenic pathways such as TGF-*β*, Wnt/β-catenin, and MYC, promoting liver fibrosis, immune evasion, and cell proliferation, which collectively advance HCC progression (Yan et al., 2024). Additionally, FOXA factors facilitate HBV transcription, sustaining viral replication and chronic infection. FOXA1 also targets PIK3R1, inhibiting the PI3K/Akt signalling pathway and thereby reducing HCC cell proliferation, migration, and invasion (He et al., 2017). The enrichment of FOXA1/2 binding sites in HBV-infected hepatocytes underscores their role in viral persistence and oncogenesis (Nevola et al., 2023).

Hepatocyte nuclear factor 4 alpha (HNF4α), another liver-specific PTF, is disrupted by HBV and implicated in liver cancer progression (Chen et al., 2024; Kotulkar et al., 2023). HNF4*α* regulates hepatocyte differentiation, metabolic homeostasis, and liver-specific gene expression, controlling genes involved in lipid metabolism, glucose regulation, and detoxification (Huck et al., 2021). Normally, HNF4α prevents oncogenic transformation by maintaining a differentiated hepatocyte state (Teeli et al., 2021). In HBV-infected hepatocytes, HNF4α competes with SOX9 to bind the EnhII/Cp region of the HBV genome, forming a feedback loop in viral replication (Yang et al., 2020). While overexpression of HNF4α reduces HBV replication by activating the NF-κB pathway and decreasing viral protein production, HBV disrupts HNF4α function, driving hepatocyte dedifferentiation and increased proliferation associated with early hepatocarcinogenesis (Teeli et al., 2021; Zhao et al., 2012).

Activator Protein-1 (AP-1), a transcription factor complex composed of c-Fos and c-Jun, is another target of HBV that enhances chromatin accessibility (Song et al., 2023). HBV infection sustains AP-1 activation, which is associated with increased expression of fibrosis markers and liver disease progression, ultimately heightening cancer risk. The interaction of HBx with AP-1 upregulates inflammatory and survival genes, exacerbating chronic liver inflammation and malignant transformation (Yang et al., 2019). Persistent AP-1 activity, mediated by signalling pathways involving SIRT1, Jab1, and JNK/c-Yun, amplifies HBV replication and fosters an environment conducive to HCC development (Park et al., 2020; Tanaka et al., 2025).

Beyond these transcription factors, HBV exploits inflammatory regulators such as STAT3 and NF-κB to sustain chronic immune activation and oncogenesis (Zakeri et al., 2024). Persistent activation of STAT3 and NF-κB, which are essential for cytokine signalling and immune responses, drives inflammation, fibrosis, and cirrhosis—major precursors to HCC (Zhao et al., 2020). HBV proteins, particularly HBc antigen, enhance chromatin binding of STAT3 and NF-κB, supporting the transcription of genes involved in cell survival, proliferation, and immune evasion (Jiang et al., 2021). Additionally, HBx-mediated activation of NF-κB increases inflammatory cytokines like IL-6 and TNF-α, perpetuating a pro-inflammatory environment conducive to hepatocarcinogenesis (Sivasudhan et al., 2022). Chronic NF-κB activation in HBV-related HCC is linked to poorer prognoses and increased tumor burden, underscoring the role of inflammation in HBV-driven oncogenesis (Sivasudhan et al., 2022).

## Chromatin accessibility and epigenetic reprogramming in HBV-related HCC

HBV utilizes its ability to manipulate host chromatin structure as a pivotal strategy for viral replication and carcinogenesis (Levrero and Zucman-Rossi, 2016). Through alterations in chromatin accessibility and epigenetic regulation, HBV promotes persistent infection, immune evasion, and oncogenic transformation (Levrero and Zucman-Rossi, 2016).

HBV integrates its DNA into the host genome, predominantly targeting actively transcribed, open chromatin regions (Figure 2). These regions, associated with actively expressed genes, create a favourable environment for viral replication (Péneau et al., 2022). HBV DNA integration can profoundly affect host gene expression, leading to the activation or silencing of genes critical for cell proliferation, survival, and differentiation (Péneau et al., 2022). Certain genomic loci are preferentially targeted by HBV, particularly those linked to cancer-related genes, and are observed more frequently in tumours than in non-tumor liver tissues (Péneau et al., 2022).

Key hotspots for integration include telomerase reverse transcriptase (*TERT*), mixed lineage leukaemia 4 (*MLL4*), cyclin E1 (*CCNE1*), *CCNA2*, *aryl-hydrocarbon receptor repressor* (*AHRR*), and tumor *protein p53 binding protein 1* (*TP53BP1*) (Péneau et al., 2022). Integration at the *TERT* locus, influenced by viral enhancers, drives overexpression of telomerase, contributing to cellular immortality, a hallmark of cancer cells (Péneau et al., 2022). Integration at the *MLL4* locus disrupts transcription factors involved in cell differentiation, promoting oncogenesis (Dong et al., 2024). At the *CCNE1* locus, integration induces cyclin E1 overexpression, enabling uncontrolled cell cycle progression—a critical step in carcinogenesis (Caldon and Musgrove, 2010). Integration at *TP53BP1* impairs DNA damage response pathways, disrupting *p53* tumor suppressor functions and driving genomic instability (Péneau et al., 2022). Clinically, HCC with a high number of HBV insertions is associated with younger patients and poorer prognoses (Péneau et al., 2022). These integration events often coincide with structural chromatin changes, including disrupted topology, gene fusions, enhancer hijacking, and abnormal oncogene activation.

HBV integration alters the three-dimensional chromatin architecture, leading to chimeric gene formation and enhancer hijacking, both of which contribute to aberrant transcription of oncogenes (Rosenkranz, 2023). Gene fusions arise from the combination of viral and host genes at integration sites, producing fusion proteins with oncogenic properties (Rosenkranz, 2023). HBV also displaces host enhancers, promoting inappropriate activation of genes associated with tumorigenesis (Levrero and Zucman-Rossi, 2016).

Epigenetic modifications, such as histone changes, are central to HBV’s manipulation of chromatin structure and gene expression (Bannister and Kouzarides, 2011). HBV infection induces specific histone acetylation and methylation changes that alter chromatin accessibility, driving transcription of both viral and host oncogenic genes (Hensel et al., 2017). The HBx protein plays a crucial role in this process, recruiting HATs like p300/CBP, which acetylate histones at lysine residues (H3K9ac and H3K27ac) to facilitate active transcription (Wang et al., 2013). Increased histone acetylation at promoter regions enhances transcription of genes critical for viral replication and oncogenesis.

HBV also activates the oncogene *c-Myc*, a key regulator of cell proliferation, through chromatin remodelling mechanisms (Jiang et al., 2021). The HBx protein activates *c-Myc* via the Ras/Raf/ERK1/2 pathway, upregulating the HSP90α promoter to enhance tumor cell invasion (Jiang et al., 2021). Additionally, HBx recruits HATs to oncogene promoters, increasing chromatin accessibility and driving the expression of cell cycle regulators necessary for uncontrolled proliferation (Haery et al., 2015). The widespread histone acetylation induced by HBx at oncogenic loci fosters hepatocyte proliferation and resistance to apoptosis (Rajan et al., 2020).

HBx-driven epigenetic reprogramming, particularly through activation of oncogenes like *c-Myc*, is integral to early liver tumor development (Rajan et al., 2020). HBx enhances *c-Myc* expression by promoting histone acetylation, increasing chromatin accessibility at oncogenic loci. Enrichment of H3K27ac, a marker of active enhancers, in HBV-infected liver cells drives transcription of genes involved in cell cycle regulation and survival (Andrisani, 2021). Conversely, HBx recruits histone methyltransferases like EZH2 and SUV39H1 to introduce repressive marks (H3K27me3 and H3K9me3), silencing key tumor suppressor genes (Yang et al., 2020; Chen et al., 2018). For example, EZH2-mediated repression of *p16INK4A*, *Rb*, and *PTEN* contributes to unchecked cell cycle progression and HBV-driven oncogenesis (Mui et al., 2017).

DNA methylation at CpG islands represents another epigenetic alteration linked to HBV infection. Elevated methylation levels at tumor suppressor gene promoters, including *CDKN2A* encoding *p16*, contribute to transcriptional silencing and hepatocarcinogenesis (Song et al., 2014; Wong et al., 2019). Hypermethylation of *CDKN2A* suppresses its role in regulating the G1-to-S phase cell cycle transition, enhancing cellular survival and tumor progression (Casciano et al., 2012). Similarly, methylation of *SOCS1*, which negatively regulates JAK–STAT signalling, heightens STAT3 activation, promoting cell proliferation and survival (Boosani and Agrawal, 2015). Other genes, such as *APC*, *GSTP1*, and *RASSF1A*, also undergo methylation-induced silencing, further driving HBV-associated HCC (Niller et al., 2012; Rongrui et al., 2014).

## Hepatitis c virus and HCC

### HCV virology

HCV is a hepatotropic, positive-sense, single-stranded RNA oncogenic virus classified within the *Flaviviridae* family and the *Hepacivirus* genus (Krump and You, 2018). It is a key etiological agent responsible for HCC, as its progressive infection, chronic liver inflammation, and cirrhosis, especially when untreated drive disease advancement (Krump and You, 2018; Virzì et al., 2018; Fiehn et al., 2024). The progression to HCC is attributed to persistent inflammation, viral protein expression, oxidative stress, and dysregulated signalling pathways, all contributing to genomic instability (Virzì et al., 2018). Approximately 80% of HCV infections become chronic, with 15–30% progressing to cirrhosis within two decades(Fiehn et al., 2024; Zhao et al., 2021). For patients with cirrhosis, the annual risk of developing HCC ranges between 1–4% (Fiehn et al., 2024; Pan et al., 2024; Khullar and Firpi, 2015). Globally, about 71 million people are infected with HCV, and 3–4 million new cases occur annually, highlighting its significant impact on public health (Pan et al., 2024).

HCV is a small, spherical virus approximately 50 nm in diameter. It is enveloped by a lipid membrane embedded with glycoproteins E1 and E2, which are critical for viral entry into host cells (Dearborn and Marcotrigiano, 2020). The HCV genome spans 9.6 kb and comprises a single open reading frame (ORF) flanked by untranslated regions (UTRs) at both ends (Romero-López and Berzal-Herranz, 2020). Upon infection, the ORF is translated into a large polyprotein (~3,000 amino acids), which is cleaved into structural proteins (Core, E1, E2, p7) and non-structural (NS) proteins (NS2, NS3, NS4A, NS4B, NS5A, NS5B) (Ashfaq et al., 2011; Dubuisson, 2007). These proteins play vital roles in viral replication, immune evasion, and pathogenesis (Ashfaq et al., 2011; Dubuisson, 2007).

The virus enters hepatocytes by binding to host cell receptors such as CD81, SR-B1, CLDN1, and OCLN, followed by internalization through clathrin-mediated endocytosis. Once inside the host cell, the viral genome is translated into a polyprotein, which is subsequently cleaved by viral and host proteases. Viral replication occurs on modified endoplasmic reticulum (ER) membranes, forming a specialized structure known as the membranous web (Sasvari and Nagy, 2010; Wolff et al., 2020). The RNA polymerase *NS5B* synthesizes a complementary negative-strand RNA, which serves as a template for producing new positive-strand RNAs (Sasvari and Nagy, 2010; Wolff et al., 2020). These RNAs are assembled into virions with core proteins and enveloped glycoproteins (E1 and E2) before being released through the host’s secretory pathway, often associated with very low-density lipoproteins (VLDL) (Vieyres et al., 2014).

HCV does not integrate its genome into the host DNA (Schinzari et al., 2015). However, it induces oncogenesis by altering chromatin accessibility and modulating epigenetic mechanisms via interactions with host PTFs and epigenetic modifiers (Morselli and Dieci, 2022). HCV proteins such as NS5A and Core are known to interact with histone-modifying enzymes, leading to changes in histone acetylation, DNA methylation, and gene expression (Żychowska et al., 2024).

### HCV-mediated regulation of PTFS

HCV significantly contributes to oncogenesis by exploiting PTFs such as FoxA1, FoxA2, HNF4α, STAT3, NF-κB, and c-Myc (Żychowska et al., 2024). These PTFs play a crucial role in maintaining an open chromatin structure, enabling liver-specific gene expression. Through this epigenetic regulation, HCV promotes hepatic gene reprogramming, viral persistence, immune evasion, chronic inflammation, and oncogenesis. Furthermore, HCV disrupts the expression of regulatory RNA molecules such as microRNAs (e.g., *miR-122*) and long non-coding RNAs (e.g., MALAT1), modifying host transcriptional networks without the need for direct genome integration (Liu et al., 2024; Okushin et al., 2021).

**Figure 3:**
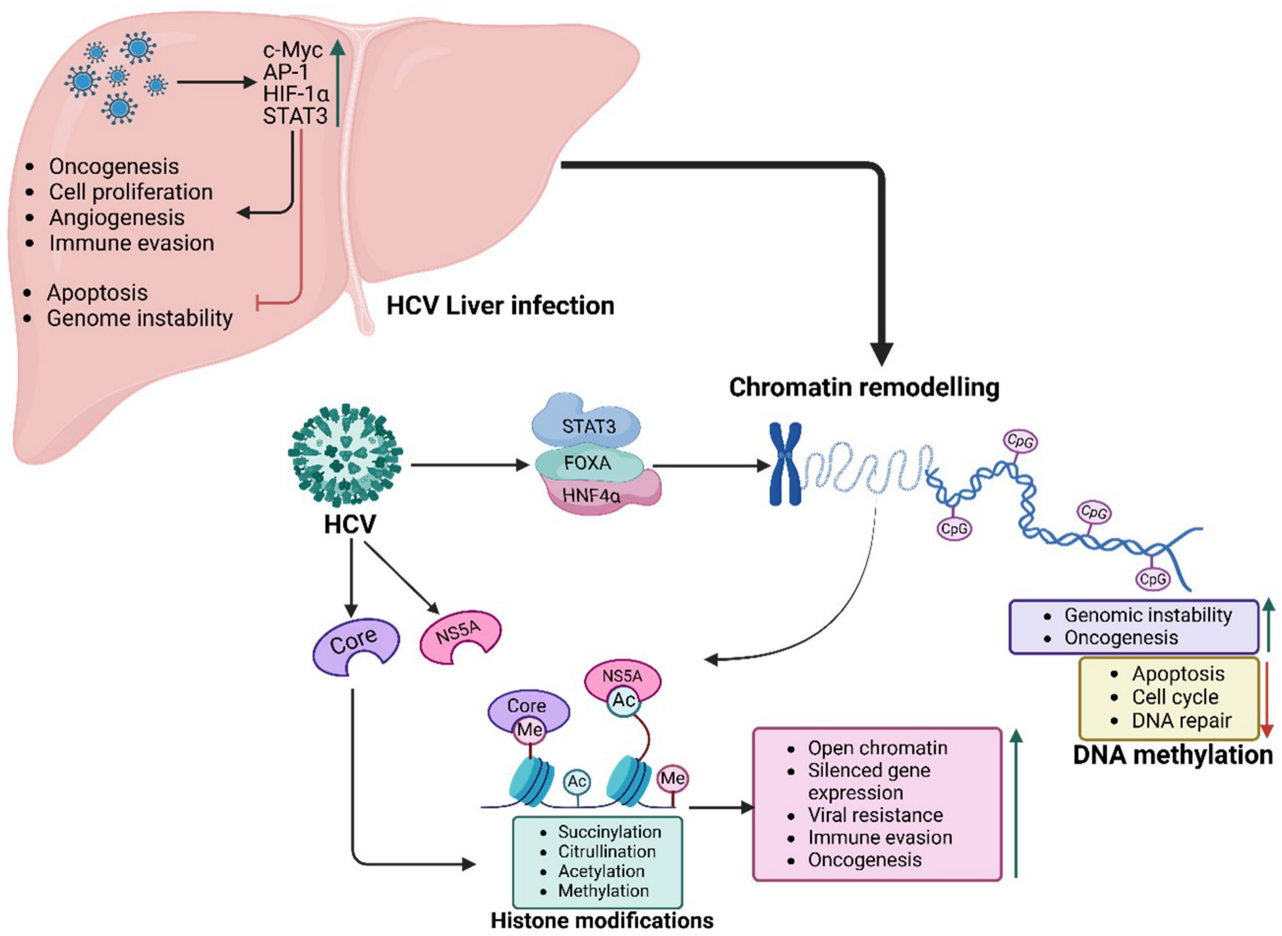
HCV-mediated PTFs regulation, chromatin remodelling and epigenetic reprogramming, and role in oncogenesis. HCV proteins Core and NS5A drive HCC by exploiting PTFs like FOXA1, FOXA2, HNF4α, STAT3, NF-κB, and c-Myc and altering epigenetic mechanisms (e.g., H3K27ac marks), including histone modifications, DNA methylation, and chromatin remodelling. These changes, coupled with circadian disruptions, activate oncogenes, silence tumor suppressors, and sustain tumorigenic potential, even after viral clearance.

HCV’s Core and NS5A proteins interact with FOXA1 and FOXA2, altering their normal functions and driving aberrant gene expression (Figure 3). This disruption leads to the upregulation of pro-oncogenic genes such as AFP and components of the TGF-*β* signalling pathway, driving processes like fibrosis, epithelial-mesenchymal transition (EMT), and tumor progression. By reshaping chromatin, HCV establishes a landscape favourable to viral replication while priming hepatocytes for malignant transformation, accelerating the progression of liver disease and promoting a tumor-supportive microenvironment (Żychowska et al., 2024).

As observed with HBV, HNF4α acts as a central regulator of hepatocyte identity, differentiation, and metabolic homeostasis (Trauner and Halilbasic, 2011). In HCV-infected cells, the virus suppresses HNF4α expression and activity, reducing chromatin accessibility and driving hepatocyte dedifferentiation (Amicone and Marchetti, 2018). This reprogramming enhances vulnerability to oncogenic transformation while fostering metabolic disruptions, inflammation, and genomic instability, hallmarks of HCV-induced oncogenesis (Amicone and Marchetti, 2018). Continuous suppression of *HNF4α* activity during chronic HCV infection is associated with more aggressive tumor phenotypes and poorer clinical outcomes in HCC patients (Vallianou et al., 2016).

In response to HCV infection, oncogenic transcription factors such as c-Myc, AP-1, HIF-1α, and STAT3 become activated, promoting uncontrolled cell proliferation, angiogenesis, and immune evasion (Vishnoi et al., 2020). The viral protein NS5A plays a key role in activating c-Myc, recruiting HATs to specific gene loci. This epigenetic remodelling upregulates genes involved in cell cycle progression, ribosome biogenesis, and nucleotide metabolism, driving unchecked hepatocyte proliferation. NS5A also interacts with the AP-1 transcription factor complex, inducing chromatin remodelling at genes linked to inflammation and oncogenesis. Sustained activation of c-Myc and AP-1 disrupts regulatory networks, enhancing oncogenic transformation, apoptosis resistance, and genomic instability, thus accelerating HCC progression (Vishnoi et al., 2020).

Chronic inflammation is a hallmark of HCV pathogenesis and serves as a driving factor for liver fibrosis, cirrhosis, and tumorigenesis (Hoshida et al., 2014). Transcriptional regulators such as STAT3 and NF-κB play critical roles in shaping the chromatin landscape of immune response and survival genes (Kaszycki and Kim, 2025). Persistent activation of STAT3 and NF-κB during HCV infection promotes the expression of genes involved in cytokine signalling, anti-apoptotic pathways, and immune evasion (Virzì et al., 2018). These factors remodel chromatin at loci associated with fibrosis and survival, creating an epigenetic environment that expedites the progression from chronic liver disease to HCC (El Taghdouini et al., 2015).

### Chromatin accessibility and epigenetic reprogramming in HCV-related HCC

HCV disrupts host pTF regulation, significantly altering the epigenetic landscape to enhance viral replication and drive hepatocyte transformation (Figure 3). These changes contribute to chronic liver disease, fibrosis, and HCC (Okushin et al., 2021; Yuan et al., 2025). Epigenetic reprogramming in HCV infection is largely mediated by histone modifications, which influence chromatin accessibility and gene expression (Hlady et al., 2022).

HCV proteins, particularly NS5A and Core, interfere with chromatin remodelling factors, causing widespread epigenetic reprogramming and abnormal gene expression (Çevik et al., 2017). These viral proteins recruit HATs like p300 and CBP, leading to increased acetylation of histone H3 and H4, which enhances chromatin accessibility at oncogenic loci (Gruber et al., 2019; Chen et al., 2022). Concurrently, HCV induces HDACs, resulting in hypoacetylation that silences tumor suppressor genes, fostering malignant transformation (Domovitz and Gal-Tanamy, 2021). Dysregulated histone methylation further exacerbates oncogenesis, as HMTs and HDMs are either recruited or inhibited by HCV, leading to silencing of tumor suppressor genes or activation of oncogenic pathways (Zeisel et al., 2021). Additionally, the virus alters nucleosome remodelling and histone variant deposition, reshaping chromatin to sustain viral replication while promoting genomic instability, inflammation, and uncontrolled cell proliferation, creating a carcinogenic microenvironment (Lieberman, 2016).

HCV-induced disruptions extend to chromatin remodelling complexes, essential for nucleosome positioning and gene regulation (Pietropaolo et al., 2021). The virus targets ATP-dependent chromatin remodelers, including the SWI/SNF and ISWI complexes, to manipulate host transcriptional programs, ensuring its persistence and oncogenesis (Pietropaolo et al., 2021). Disruption of these complexes silences tumor suppressor genes and activates pro-oncogenic pathways (Hu et al., 2021). For example, HCV’s interference with BRG1 and BRM components shifts gene expression toward pro-inflammatory and oncogenic states, amplifying NF-κB and STAT3 signalling, which promotes fibrosis, inflammation, and genomic instability, accelerating HCC progression (Tonon, 2016; Pfefferlé and Vallelian, 2024). Several analyses identified genes like *MORF4L1*, *HDAC1*, *VPS72*, and *RUVBL2* as key ATP-dependent chromatin remodelling-related genes (ACRRGs) influencing HCC prognosis (Xu et al., 2021). Functional studies confirmed that *MORF4L1* enhances cancer stemness through Hedgehog signalling, highlighting its role in tumor growth and metastasis (Xu et al., 2021).

Emerging research emphasizes the critical role of histone post-translational modifications including succinylation, citrullination, and acetylation in regulating chromatin architecture and influencing tumor growth, metastasis, and metabolic reprogramming (Wang et al., 2025). Genome-wide analyses reveal that HCV infection induces changes in histone marks, such as H3K4me3 and H3K9ac, which are linked to oncogenic pathways (Perez et al., 2019). Even after achieving a sustained virologic response (SVR) with direct-acting antivirals (DAAs), HCV leaves behind persistent epigenetic changes. These alterations, particularly in H3K27ac, remain beyond viral clearance and continue to drive oncogenic processes, contributing to the elevated risk of HCC post-SVR (Perez et al., 2019).

Studies also explore how HCV reshapes the 3D structure of the host genome through chromatin-organizing factors like CTCF and cohesin, which influence both viral and cellular genome configurations. These structural changes can promote a persistent pro-oncogenic epigenetic landscape even after viral clearance (Kim and Lieberman, 2024). Additionally, HCV infection disrupts the circadian regulation of gene expression in the liver, disturbing chromatin remodelling pathways and promoting a pro-tumorigenic environment (Mukherji et al., 2024). Such disruptions reveal how HCV exploits circadian mechanisms for chronic infection and oncogenic transformation.

Aberrant DNA methylation is another hallmark of HCV-induced epigenetic reprogramming. HCV proteins, particularly Core and NS5A, modulate DNMTs, resulting in hypermethylation of tumor suppressor gene promoters and hypomethylation of oncogenes. These changes promote genomic instability, silencing genes involved in cell cycle regulation, apoptosis, and DNA repair, while activating proto-oncogenes to enhance tumor progression (Cheng et al., 2021). Methylation patterns associated with HCV-induced cirrhosis and HCC serve as biomarkers for early detection and risk stratification. Distinct methylation profiles in HCV-driven HCC compared to HBV-driven HCC highlight virus-specific mechanisms underlying oncogenesis, offering opportunities for precision medicine approaches (Kim and Lieberman, 2024).

## Human papillomavirus and cervical cancer

### HPV virology

HPV, a circular, double-stranded DNA (dsDNA) virus from the *Papillomaviridae* family, is a small, non-enveloped pathogen primarily infecting epithelial tissues (Liu et al., 2021). Comprising around 52 genera, HPV is highly oncogenic and epitheliotropic, with a genome of approximately 8 kb. It is a major cause of cervical cancer, anogenital malignancies, specific head and neck squamous cell carcinomas (HNSCCs), and nasopharyngeal cancer (NPC), accounting for approximately 70% of cervical cancer cases. Its genome is divided into three regions: the early (*E* genes, including the oncogenes E6 and E7 essential for replication and oncogenesis), the late (L genes responsible for structural proteins), and the upstream regulatory region (URR) that governs transcription and replication. The long control region (LCR) harbours regulatory sequences essential for controlling viral gene expression (Kurvinen et al., 2000).

HPV is a sexually transmitted virus categorized into low, medium, and high-risk strains, with the International Agency for Research on Cancer (IARC) identifying 12 high-risk genotypes. Among its over 200 recognized types, low-risk strains like HPV-6 and HPV-11 are associated with benign conditions such as genital warts, whereas high-risk strains, including HPV-16 and HPV-18, are strongly linked to cervical, anogenital, and oropharyngeal cancers. High-risk strains significantly contribute to various malignancies, such as cervical, penile, and head and neck cancers (Kidd et al., 2017).

HPV infects basal epithelial cells through microabrasions in the skin or mucosa. Infections with high-risk HPV strains involve integration of the viral genome into the host DNA, which disrupts normal cell cycle regulation. Key viral proteins, *E6* and *E7*, drive oncogenesis by deactivating tumor suppressors like *p53* and *pRb*, resulting in unchecked cell proliferation and immune evasion (Yim and Park, 2005). Persistent high-risk HPV infections significantly increase the risk of cancer, causing nearly all cervical cancers, 90% of anal cancers, and substantial proportions of vaginal and oropharyngeal cancers (Egawa, 2023). The virus evades immune detection by downregulating antigen presentation and interfering with interferon signalling, thereby prolonging infections (Yim and Park, 2005).

Preventive strategies have been highly effective in reducing HPV-associated diseases. Vaccines such as Gardasil and Cervarix target high-risk HPV types, significantly decreasing cervical cancer rates (Cheng et al., 2020). Early detection methods, including Pap smears and HPV DNA testing, are essential for identifying precancerous lesions. Treatment options such as surgical removal, cryotherapy, and immune-modulating therapies help manage HPV-related conditions. However, the absence of specific antiviral therapies for HPV remains a limitation in combating the virus (Pathak et al., 2022).

### HPV-mediated regulation of PTFS

**Figure 4:**
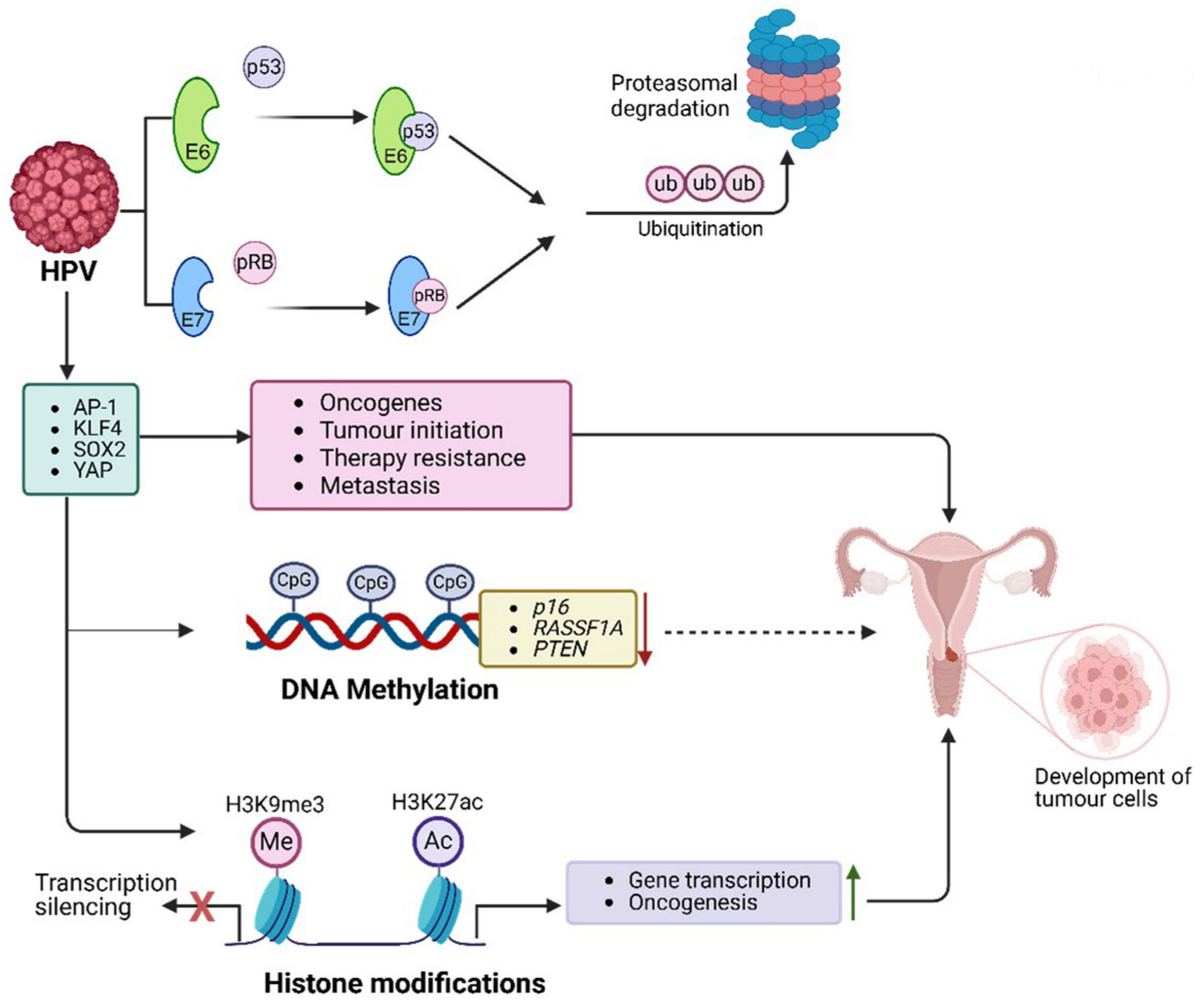
HPV-mediated PTFs regulation, chromatin remodelling and epigenetic reprogramming, and role in oncogenesis. HPV drives cancer through epigenetic alterations. Viral proteins E6 and E7 modify host chromatin to activate oncogenes and silence tumor suppressor genes (e.g., *p16, RASSF1A*), disrupting cell cycle regulation. E7 degrades *pRB*, releasing E2F transcription factors, enhancing survival and proliferation genes (e.g., *MYC*, *CDK2*). HPV manipulates PTFs like AP-1, KLF4, YAP/TEAD, and SOX2 to sustain viral oncogene transcription, remodel chromatin, and drive malignancy. These changes, combined with chromatin remodelling, histone modifications (e.g., H3K27ac), and DNA methylation, lead to genomic instability and promote cervical, anogenital, and oropharyngeal cancers.

HPV utilizes various host PTFs to regulate its oncogenic processes, transitioning from latent infection to active viral replication and contributing to malignancy (Figure 4). Among these, AP-1 plays a pivotal role by binding to LCR enhancer elements and activating early HPV genes, including E6 and E7 (Liu et al., 2002; Yee, 2013). AP-1 remodels chromatin by recruiting p300/CBP to induce H3K27 acetylation (H3K27ac), enhancing chromatin accessibility and enabling transcription of the E6/E7 promoters. This process sustains viral oncogene expression and promotes malignancy (Wang et al., 2025; Yee, 2013). Furthermore, AP-1 activates host oncogenes such as MYC and cyclin E/cyclin-dependent kinase (CDK), facilitating cell cycle progression and proliferation. Its persistent activity in HPV-driven cancers reinforces oncogenesis (McLaughlin-Drubin and Münger, 2009).

Krϋppel-like factor 4 (KLF4) also acts as an HPV PTF, enabling transcription by decondensing compacted chromatin (Tiwari et al., 2013; Yang and Zheng, 2012). In HPV-positive cancers, KLF4 promotes viral oncogene transcription by targeting silenced genomic regions, generating accessible chromatin structures (Gunasekharan et al., 2016). Upregulated through post-transcriptional and post-translational modifications, KLF4 affects specific target genes, including *TCHHL1*, *VIM*, *ACTN1*, and *POT1*, driving tumor growth by sustaining cell cycle progression, a proliferative state, and resistance to differentiation signals in HPV-infected epithelial cells (Gunasekharan et al., 2016). Overexpression of KLF4, mediated by E6 and E7, is crucial for HPV genome amplification and late gene expression. Silencing *KLF4* using shRNAs disrupts these processes (Gunasekharan et al., 2016). Additionally, KLF4 protein collaborates with chromatin remodelers like SWI/SNF to enhance accessibility, reprogram endothelial enhancers, and establish transcriptional networks supporting oncogenic gene expression (Moonen et al., 2022). Enhancer-promoter loops involving KLF4 regulate genes like *BMPR2*, *SMAD5*, and *DUSP5*, stabilizing chromatin and sustaining *E6*/*E7* oncogene expression (Gunasekharan et al., 2016).

The HPV E6/E7 oncoproteins also target Yes-associated protein 1 (YAP1) and large tumor suppressor kinases 1 and 2 (LATS1/2), key regulators of the Hippo signalling pathway (Blakely et al., 2024). By degrading YAP1 and LATS1/2, E6/E7 block YAP phosphorylation, promoting its nuclear translocation and interaction with TEAD transcription factors. The activated YAP/TEAD complexes increase chromatin accessibility, facilitating transcription of genes essential for cell survival, proliferation, and apoptosis resistance (Blakely et al., 2024; Zhao et al., 2023). This results in the upregulation of oncogenic targets such as CCND1, which drives the G1/S cell cycle transition, and BIRC5 (Survivin), an anti-apoptotic protein. Persistent YAP/TEAD activation in HPV-associated cancers underscores the Hippo pathway as a therapeutic target (Chiou et al., 2003; Montalto and De Amicis, 2020).

Another key factor, SOX2, is upregulated by HPV infection to maintain stemness and self-renewal. Elevated SOX2 expression in HPV-positive cancers contributes to tumor initiation, therapy resistance, and metastasis by promoting cancer stem-like phenotypes (Mamun et al., 2020). SOX2-positive cells show increased tumorigenic potential and resistance to differentiation cues, driving disease progression (Mamun et al., 2020). SOX2 interacts with chromatin modifiers like HATs and chromatin remodelers, facilitating epigenetic reprogramming in HPV-infected epithelial cells (Soto et al., 2017). Moreover, it collaborates with transcription factors like AP-1, KLF4, and TEAD/YAP to enhance chromatin accessibility at oncogene loci, further sustaining the malignant transformation (Mamun et al., 2020).

Overall, the above evidence underscores the interconnected roles of AP-1, KLF4, YAP1, and SOX2 in HPV-driven malignancies as displayed in Figure 4, demonstrating their contributions to chromatin remodelling, viral gene transcription, and oncogenic progression.

### Chromatin accessibility and epigenetic reprogramming in HPV-related cervical cancer

HPV demonstrates its oncogenic potential by interacting with host chromatin, reprogramming chromatin architecture, and regulating gene transcription (Figure 4). These activities lead to cell cycle disruption and immune evasion (McLaughlin-Drubin and Münger, 2009; Doorbar et al., 2012). The virus relies on transcriptional regulators like P97 and P670 to manipulate host chromatin for viral replication and oncogenesis (Castro-Oropeza and Piña-Sánchez, 2022). Additionally, HPV may play a role in the metastasis of aggressive breast cancers by activating specific transcription factors (Ghoreshi et al., 2023). Despite lacking intrinsic chromatin-modifying enzymes, HPV reprograms host epigenetic machinery through episomal maintenance and genome integration, ultimately enhancing viral transcription and oncogenesis.

During infection, HPV targets the basal layer of stratified epithelia, maintaining its genome as a low-copy episome in basal cell nuclei (reviewed in 146). Episomal maintenance is essential for viral persistence and stable replication alongside host DNA during cell division. Viral proteins E1 and E2 facilitate replication and partitioning of the viral genome to daughter cells, enabling HPV to persist without immediate genome integration (McBride, 2017). However, the integration of viral DNA into the host genome represents a pivotal step in malignancy progression. This process disrupts normal chromatin architecture and gene regulation, potentially inactivating or aberrantly expressing host genes (Zhang et al., 2016). For instance, insertional mutagenesis may disable tumor suppressor genes, driving uncontrolled proliferation (Zhang et al., 2016). Furthermore, integration introduces binding sites for host transcription factors, reorganizing local chromatin structure and forming new topologically associating domains (TADs), which aberrantly regulate gene expression. These changes contribute to oncogenesis by activating oncogenes or repressing tumor suppressor genes (Zhang et al., 2016).

HPV preferentially integrates into common fragile sites (CFSs), genomic regions prone to breakage and instability under replication stress (Warburton et al., 2021). Such integration disrupts chromatin folding, promotes genomic instability, and fosters chromosomal rearrangements and mutations—hallmarks of cancer progression. While episomal HPV genomes maintain controlled viral gene expression (Durzynska et al., 2017), integration results in the loss of regulatory elements, leading to unchecked expression of E6 and E7 oncoproteins (Yeo-Teh et al., 2018). This dysregulation bypasses cellular checks, sustaining oncogene expression, promoting proliferation, and inhibiting tumor suppressors. Integration also induces DNA methylation at tumor suppressor gene promoters, silencing them while opening chromatin at oncogene loci, enhancing expression of genes involved in cell cycle progression and survival (Soto et al., 2017). These epigenetic changes create a permissive environment for cancer progression (Mac and Moody, 2020).

HPV oncoproteins E6 and E7 play central roles in epigenetic reprogramming by influencing chromatin remodelling, histone modifications, and DNA methylation (Sen et al., 2018). E6 promotes p53 degradation via ubiquitination mediated by E6-associated protein (E6AP), impairing p53-dependent chromatin repression and DNA repair pathways. This drives unchecked proliferation and survival. The viral genome forms a chromatin-like structure with host histones, subject to histone modifications. While H3K27ac enhances early gene transcription, promoting oncogenesis, H3K9me3 is linked to transcriptional silencing and latency (Mac and Moody, 2020). Chromatin remodelers like HATs and HDACs dynamically regulate these marks, controlling viral DNA accessibility (Mac and Moody, 2020). E6 of high-risk HPV further activates oncogenes like EGFR and c-MET by destabilizing histone demethylase KDM5C (Chen et al., 2018). Inhibition of Sp1 impacts active histone marks and HPV-host chromatin interactions, reducing oncogene expression and enhancing immune checkpoint gene expression (Cao et al., 2024).

E7 targets retinoblastoma protein (pRB) for proteasomal degradation, releasing E2F transcription factors and driving chromatin opening at cell cycle-related genes like CDK2, Cyclin A, and MYC (Yeo-Teh et al., 2018). This disrupts critical cell cycle checkpoints, enabling uncontrolled proliferation. E7 recruits p300/CBP histone acetyltransferases, catalysing H3K27ac, a hallmark of active enhancers, to drive the transcription of proliferation and survival genes (Wang et al., 2022). Additionally, E7 inhibits HDAC1, maintaining an open chromatin state and ensuring sustained oncogene expression (Wang et al., 2022).

E6 also promotes global DNA hypermethylation through DNMT1, silencing tumor suppressor genes such as *p16*, *RASSF1A*, and *PTEN*, while downregulating TET enzymes responsible for DNA demethylation. This leads to hypermethylated DNA, suppressing tumor suppressor genes and advancing carcinogenesis (Sen et al., 2018).

## Human t-cell leukaemia virus type 1 and ATLL

### HTLV-1 virology

HTLV-1, a member of the *Retroviridae* family, is a well-established causative agent of oncogenic and inflammatory diseases, most notably adult T-cell leukaemia/lymphoma (ATLL), a malignancy of CD4 + T cells (Eusebio-Ponce et al., 2019). ATLL is characterized by uncontrolled proliferation of infected CD4 + T cells, affecting organs such as the lymph nodes, liver, or spleen (Graham et al., 2014). It is categorized into four subtypes: acute, chronic, smouldering, and lymphoma, with the acute subtype being the most prevalent (Letafati et al., 2023). HTLV-1 has a 9 kb genome consisting of +ssRNA and encodes structural and enzymatic genes such as *gag*, *pro*, *pol*, and *env* (Azodi et al., 2017). HTLV-1 also expresses accessory genes, including the transactivator protein *Tax*, transcribed from the sense strand, which is critical for cellular transformation and transcriptional activation of the 5′ LTR promoter region (Enose-Akahata et al., 2017). In contrast, HBZ, encoded from the antisense strand, produces the HBZ protein, which counteracts Tax activities (Carcone et al., 2022).

Upon infecting host cells, HTLV-1 undergoes reverse transcription, converting its +ssRNA genome into dsDNA, which integrates into the host genome. The viral genome is flanked by long terminal repeats (LTR) at the 5′ and 3′ ends (Matsuoka and Mesnard, 2020; Martinez et al., 2019). These LTR regions, comprising U3, R, and U5 domains, regulate key processes like viral transcription, polyadenylation, and integration, ensuring efficient viral gene expression (Ernzen and Panfil, 2022).

HTLV-1-mediated T-cell malignancies, including lymphomas and leukaemia’s, result from dysregulated T-cell development and poor clinical outcomes (Andreescu, 2024). Epigenetic modifications, such as chromatin remodelling and alterations in miRNA activity, play a crucial role in transcriptional regulation of viral and host genes, including *Tax* and *HBZ* (Pietropaolo et al., 2021). These changes exploit host transcription factors such as NF-κB, AP-1, and STAT3, driving increased chromatin accessibility, tumorigenesis, immune evasion, and altered gene expression (Dong et al., 2024). The Tax protein activates oncogenes and inflammatory pathways, while HBZ represses tumor suppressor genes, ensuring continued immune evasion (Dong et al., 2024).

By reprogramming the host’s genetic and epigenetic landscapes, HTLV-1 creates conditions favourable for malignancy (Yamagishi et al., 2018). Emerging therapeutic approaches, including epigenetic regulators, NF-κB inhibitors, and immunotherapy, hold promise for managing ATLL and other HTLV-1-associated disorders (Dong et al., 2024).

### HTLV-1-mediated regulation of PTFS

**Figure 5:**
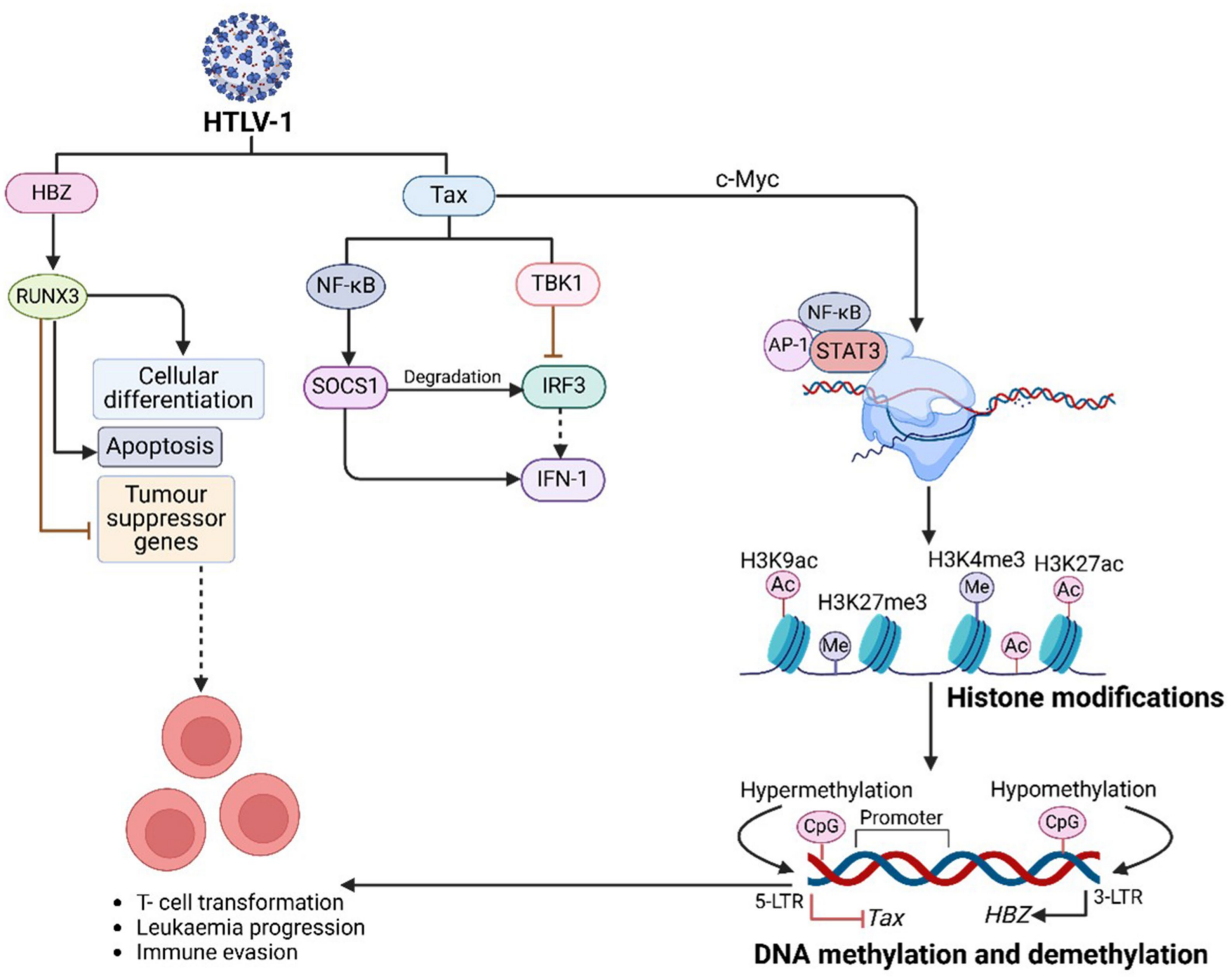
HTLV-1-mediated PTFs regulation, chromatin remodelling and epigenetic reprogramming, and role in oncogenesis. HTLV-1 + ssRNA genome integrates into active euchromatin regions, leveraging LTRs for viral gene expression. HTLV-1 promotes oncogenesis through chromatin remodelling (through, e.g., SWI/SNF, CHD, INO80), histone modifications, and DNA methylation, driven by its Tax and HBZ proteins. Tax enhances inflammation and transcription, while HBZ silences tumor suppressor genes, enabling malignant progression. Targeting these pathways with epigenetic therapies offers potential treatment for ATLL.

HTLV-1 employs PTFs to evade immune responses, promote T-cell transformation, and drive leukaemia progression (Figure 5). By manipulating host gene expression, HTLV-1 regulates its replication while avoiding immune detection (Mohanty and Harhaj, 2023). The viral protein HBZ facilitates immune evasion by leveraging the NF-κB signalling pathway. Concurrently, Tax, another key viral protein, contains nuclear localization (NLS) and nuclear export (NES) signals, enabling its movement between the nucleus and cytoplasm, where it activates NF-κB and drives viral gene expression (Mohanty and Harhaj, 2023). Mutations in Tax often render it undetectable in peripheral blood mononuclear cells (PBMCs) of ATLL-infected individuals, as Tax-expressing cells are highly immunogenic and targeted by cytotoxic T cells. Mutations in the HTLV-1 Tax protein can reduce its detectability in peripheral blood mononuclear cells (PBMCs) of individuals with ATLL. Tax-expressing cells are highly immunogenic and are actively targeted by cytotoxic T cells (Mohanty and Harhaj, 2023). Consequently, certain mutations may alter Tax’s structure or expression, enabling infected cells to evade immune surveillance and diminishing detectable Tax levels in PBMCs. However, wild-type Tax is generally considered highly immunogenic, while specific mutations can either maintain or reduce this immunogenicity depending on their impact on Tax function and recognition by the immune system (Mohanty and Harhaj, 2023). Additionally, Tax suppresses IRF3 activity by interacting with TBK1, which reduces antiviral IFN-1 responses. It also activates SOCS1 through NF-κB, leading to IRF3 degradation, dysregulated IFN-*β* secretion, and suppression of TLR4 signalling, further aiding viral immune evasion (Mohanty and Harhaj, 2023).

HBZ binds to RUNX3, a runt-related transcription factor that regulates apoptosis and differentiation. By displacing RUNX3 from tumor suppressor gene promoters, HBZ ensures tumor suppressor gene inactivation. Mutations or abnormal expression of RUNX3 are strongly associated with cancer development, highlighting the oncogenic potential of HBZ (Kulkarni et al., 2018).

The Tax protein activates *c-Myc*, a proto-oncogene that regulates transcription of cancer-related genes. This activation results in histone acetylation at oncogenic promoters such as CCND1 and E2F1 (Wallbillich and Lu, 2023). *c-Myc* interacts with key signalling pathways, including Wnt/β-catenin, JAK/STAT, MAPK, and NF-κB. By recruiting HATs like p300/CBP and TIP60, c-Myc enhances chromatin accessibility at super-enhancers, promoting aggressive malignancies such as ATLL (Sundeep et al., n.d.). Dysregulated bromodomain and extraterminal domain (BET) activity, involving BRD4, further amplifies oncogenic transcription through H3K122 acetylation. BET and *HAT* inhibitors have shown potential in targeting these pathways to combat *c-Myc*-driven leukemogenesis (Table 1) (Verbeke et al., 2025).

**Table 1:** Emerging therapeutic strategies targeting epigenetic and transcriptional dysregulation in oncogenic viruses.

Therapeutic strategy	Mechanism of action	Example agents	Clinical status	Potential applications	Reference/s
Hepatitis B virus and hepatocellular carcinoma					
Checkpoint inhibitors	Restore anti-tumour immune response in HBV-associated HCC	Anti-PD-1 (Nivolumab, Pembrolizumab)	Clinical trials	Treatment of HBV-associated hepatocellular carcinoma	Li et al. (2020), Mon et al. (2025), and Burns et al. (2021)
CRISPR-Cas9 mediated gene editing	Target HBV covalently closed circular DNA (cccDNA) to disrupt viral replication	CRISPR-Cas9	Preclinical	Directly targeting viral genome to permanently suppress HBV infection	Martinez et al. (2022), Martinez et al. (2022), and Yao et al. (2024)
DNA methyltransferase inhibitors	Reverse abnormal methylation in HBV-infected cells to restore regular gene expression	Decitabine, Azacitidine	Clinical trials	Epigenetic reprogramming to suppress viral replication and reduce liver fibrosis	Dai et al. (2024), Zeidan et al. (2022), and Jabbour et al. (2017)
Histone deacetylase (HDAC) inhibitors	Enhance histone acetylation to reactivate silenced genes and inhibit HBV replication	Vorinostat, Panobinostat	Preclinical/Clinical trials	Epigenetic reprogramming to suppress viral replication and reduce liver fibrosis	Dai et al. (2024), Pan et al. (2023), and Yang et al. (2021)
RNA-based therapies	Gene silencing or genome editing to suppress HBV-associated oncogenic drivers	siRNA, CRISPR-Cas9	Preclinical	Targeting viral or host oncogenic factors at the genetic level	Zoulim et al. (2024), Najafi et al. (2022), He et al. (2024), Bartosh et al. (2024), and Kumar et al. (2024)
HBV therapeutic vaccines	Stimulate immune response against HBV antigens to prevent progression to HCC	HBV DNA vaccines, peptide vaccines	Preclinical/Clinical trials	Immunisation strategy to reduce HBV-associated carcinogenesis	Mahmood et al. (2023), Kramvis et al. (2023), and Cargill and Barnes (2021)
Hepatitis C virus and hepatocellular carcinoma					
Histone deacetylase (HDAC) inhibitors	Restore gene expression by promoting histone acetylation	Vorinostat, Panobinostat	Preclinical/Clinical trials	Reactivation of tumour suppressor genes, reducing HCC progression	Lachenmayer et al. (2012), Sanaei and Kavoosi (2021), and Banerjee et al. (2018)
DNA methyltransferase inhibitors	Reverse aberrant DNA methylation to restore normal gene expression	Decitabine, Azacitidine	Clinical trials	Epigenetic reprogramming to inhibit oncogenic pathways	Babar et al. (2022)
Direct-acting antiviral (DAA)-based targeted therapy	Inhibit key viral proteins: NS3/4A protease, NS5A, and NS5B polymerase	Sofosbuvir (NS5B inhibitor); Velpatasvir; Glecaprevir/Pibrentasvir	Approved	Treatment of acute/chronic HCV, HIV/HCV co-infection, cirrhosis, post-transplant HCV	Cotter and Jensen (2019), Horner and Naggie (2015), Kimberlin (2023), and Lim and Gallay (2014)
Next-gen DAA (in development)	Targeting drug-resistant HCV variants, improved pharmacokinetics	Bemnifosbuvir (AT-527)	Clinical trials	Refractory cases, improved options for special populations	Zhou et al. (2024)
Bromodomain (BET) inhibitors	Disrupt interaction of BET proteins with acetylated histones, suppressing oncogenic transcription	JQ1, OTX015	Preclinical/Clinical trials	Targeting Myc-driven transcription in HCV-associated HCC	Stathis and Bertoni (2018) and Coudé et al. (2015)
STAT3, Myc, HIF-1α pathway inhibitors	Block oncogenic transcription factor signalling	Stattic (STAT3 inhibitor), Myc inhibitors (Omomyc)	Preclinical/Clinical trials	Inhibiting tumour-promoting transcriptional activity	Xu et al. (2021) and Lee and Cheung (2019)
RNA-based therapies	Gene silencing or genome editing to suppress HCV-associated oncogenic drivers	siRNA, CRISPR-Cas9	Preclinical	Targeting viral or host oncogenic factors at the genetic level	Wu et al. (2020)
Checkpoint Inhibitors	Restore immune response against HCV-associated HCC	Anti-PD-1 (Nivolumab), Anti-CTLA-4 (Ipilimumab)	FDA-approved	Enhancing anti-tumour immunity in HCC	Kudo (2024)
Therapeutic Vaccines	Stimulate immune response against HCV proteins to prevent progression to HCC	HCV peptide vaccines, DNA vaccines	Preclinical/Clinical trials	Immunisation strategy to reduce HCV-associated carcinogenesis	Mackesy-Amiti et al. (2024) and Czarnota et al. (2024)
Human papillomavirus and cervical cancer					
HPV therapeutic vaccines	Stimulate immune response against HPV E6 and E7 oncoproteins	HspE7, VGX-3100	Preclinical/Clinical trials	Treatment of cervical intraepithelial neoplasia (CIN) and cervical cancer	Trimble et al. (2015), Bhuyan et al. (2021), and Lo Cigno et al. (2024)
Checkpoint inhibitors	Block immune checkpoint proteins to enhance T-cell response against HPV + tumours	Anti-PD-1 (Nivolumab, Pembrolizumab)	Clinical trials	Treatment of HPV-associated head and neck cancers	Zabeti Touchaei and Vahidi (2024), Lee and Allen (2021), and Zielińska et al. (2025)
Epigenetic modifiers	Reactivate tumour suppressor genes by modifying histone acetylation or DNA methylation	Azacytidine, Vorinostat	Clinical trials	Targeting epigenetic dysregulation in HPV-driven cancers	Letafati et al. (2025), Cheng et al. (2019), and Patnaik et al. (2023)
RNA-based therapies	Gene silencing or genome editing to suppress HPV oncogenes	siRNA, CRISPR-Cas9	Preclinical	Direct inhibition of HPV E6 and E7 oncogene expression	Inturi and Jemth (2021), Pal and Kundu (2019), Shiri Aghbash et al. (2023), and Shanmugam et al. (2025)
Targeted therapy	Block molecular pathways involved in HPV-driven tumour progression	PI3K inhibitors, mTOR inhibitors	Preclinical/Clinical trials	Inhibition of HPV-related oncogenic signalling pathways	Shanmugam et al. (2025), Shan et al. (2024), and Verhees et al. (2025)
Human T-cell leukaemia virus type 1 and adult T-cell leukaemia/lymphoma					
Epigenetic modifiers	Alter gene expression without changing the DNA sequence	Azacytidine, Pralatrexate	Clinical trials	Epigenetic therapy for ATLL by restoring tumour suppressor gene function	Letafati et al. (2025), Zhang et al. (2019), and Rosenthal et al. (2023)
Checkpoint inhibitors	Restore immune response against HTLV-1-driven ATLL	Anti-PD-1 (Nivolumab, Pembrolizumab)	Clinical trials	Enhancing T-cell response against HTLV-1-associated malignancies	Ghione et al. (2018), Neuwelt et al. (2020), and Jalili-Nik et al. (2021)
Gene therapy approaches	Correct defective genes or introduce new genes to treat or prevent disease	Gene editing (CRISPR-Cas9, TALENs)	Preclinical	Potential cure for HTLV-1 infection and associated malignancies	Wang et al. (2024), Domingues et al. (2024), Li et al. (2020), and Garg et al. (2025)
Targeted therapy	Block HTLV-1-driven oncogenic pathways	NF-κB inhibitors, JAK inhibitors	Preclinical/Clinical trials	Inhibiting key signalling pathways in ATLL	Mohanty and Harhaj (2023), Hu et al. (2021), and Li et al. (2024)
Epstein–Barr virus and associated cancers					
Immunotherapy (Checkpoint inhibitors)	Enhance immune response to target EBV-infected cells	Monoclonal antibodies (Nivolumab, Pembrolizumab)	Clinical trials	Treatment of EBV-associated lymphomas and nasopharyngeal carcinoma	Liu et al. (2024), Li et al. (2022), Cai et al. (2024), Wang et al. (2023), and Soldan et al. (2022)
Histone methyltransferase inhibitors	Inhibit EZH2 to suppress viral oncogene expression	Tazemetostat	Preclinical	Reducing EBV-associated lymphomas by suppressing oncogenic transcription factors	Sarkozy et al. (2020), Julia and Salles (2021), and Nastoupil et al. (2023)
BET inhibitors	Disrupt BET protein interaction to reduce transcription of EBV oncogenes	JQ1, OTX015	preclinical/clinical trials	Targeting latent EBV-infected cells and reactivating immune responses to reduce tumor burden	Li et al. (2018) and Smith et al. (2016)
Antiviral drugs	Inhibit EBV replication to reduce viral load and tumour progression	Ganciclovir, Valganciclovir	Clinical trials	Potential adjunctive therapy in EBV-related cancers	Salnikov et al. (2024), Soldan et al. (2022), Allen et al. (2019), Yager et al. (2017), and Oumata et al. (2025)
Targeted therapy	Block specific pathways activated by EBV to prevent tumour growth	PI3K inhibitors, Hedgehog pathway inhibitors	Preclinical/Clinical trials	Disruption of EBV-driven oncogenic signalling pathways	Low et al. (2023) and Tsang et al. (2023)
Human herpesvirus 8 virus and Kaposi’s sarcoma					
Tyrosine kinase inhibitors	Inhibit tyrosine kinases to reduce tumor growth in HHV-8-associated malignancies	Imatinib	Clinical trials	Treatment of KS and other HHV-8-associated diseases	Iqbal and Iqbal (2014), Keup et al. (2023), and Koon et al. (2013)
Monoclonal antibody therapy	Target viral IL-6 to alleviate symptoms of multicentric castleman’s disease (MCD)	Anti-IL-6 (siltuximab and tocilizumab)	FDA-approved, clinical trials	Management of HHV-8-associated MCD	van Rhee et al. (2018), Lurain et al. (2018), and Gliga et al. (2021)
Epigenetic modulators (DNMT inhibitors)	Reprogram KSHV-infected cells through demethylation	Azacitidine	Preclinical	Suppressing KSHV lytic replication and oncogenesis	Okpara et al. (2024) and Naimo et al. (2021)
STAT3 pathway inhibitors	Block STAT3 signalling to inhibit KSHV-driven oncogenesis	Stattic	Preclinical	Reducing KSHV oncogenic activity and halting Kaposi’s sarcoma progression	Hopcraft et al. (2018) and Li et al. (2019)
Histone deacetylase (HDAC) Inhibitors	Promote reactivation of silenced genes and inhibit viral oncogene expression	Vorinostat, Romidepsin	Preclinical/Clinical Trials	Reducing HHV-8 progression by reactivating tumor suppressor genes	Hopcraft et al. (2018), Lu et al. (2023), and Murphy et al. (2022)
Redox disruption agents	Disrupt redox balance to inhibit HHV-8 replication and associated malignancies	Primaquine, and Resveratrol	Preclinical	Potential treatment for HHV-8-related malignancies	Gothland et al. (2023)
Virus-specific T cell therapy	Infuse HHV-8-specific T cells to target and eliminate HHV-8-infected cells	ALVR108	Clinical Trials	Treatment of HHV-8-associated diseases in immunocompromised patients	Houghtelin and Bollard (2017)
Merkel cell polyomavirus and merkel cell carcinoma					
Adjuvant therapy	Enhance immune response against MCV by modulating the tumor microenvironment	GLA-SE (TLR4 agonist)/GLA-100	Clinical Trials	Treatment of MCV-associated MCC	Bi et al. (2023) and Vandeven and Nghiem (2016)
DNA methyltransferase inhibitors	Reverse aberrant methylation in MCV-infected cells to suppress viral oncogene expression	Decitabine, azacitidine	Clinical trials	Targeting MCC by suppressing viral oncogenes	Harms et al. (2022) and Stewart et al. (2009)
Bromodomain (BET) inhibitors	Inhibit BET proteins to reduce viral transcription in MCV-associated cancers	JQ1, OTX015	Preclinical/clinical trials	Reducing viral load and tumor growth in MCV-driven cancers	Rizzitano et al. (2016), Dombret et al. (2014), and Ocana et al. (2015)
Oncolytic viral therapy	Use engineered viruses to selectively infect and destroy MCV-infected tumor cells	T-VEC (HSV-based)	Preclinical	Potential treatment for MCV-driven MCC	Conry et al. (2018), de Almeida et al. (2021), Gambichler et al. (2024), and Russell and Peng (2017)
Immune checkpoint blockade	Block inhibitory signals to enhance T-cell-mediated immune response against MCV-infected cells	Avelumab, allogeneic activated NK cell (aNK)	FDA-approved, clinical trials	Combination therapy with vaccines for MCC	Schadendorf et al. (2017) and Becker et al. (2024)
mRNA therapeutic vaccines	Deliver mRNA encoding MCV antigens to stimulate a targeted immune response	LTA mRNA	Clinical trials	Prophylactic and therapeutic vaccination for MCV-associated MCC	Frey et al. (2024) and Xu et al. (2021)

Tax induces the AP-1 transcription factor complex (c-Fos and c-Jun), remodelling chromatin at genes involved in T-cell activation. This activation promotes the expression of oncogenic genes such as *IL-2Rα* (*CD25*), a marker of ATLL proliferation (Ahmadi Ghezeldasht et al., 2023). Tax-dependent AP-1 activation drives the expression of cytokines like IL-2, IL-8, and TNF-α, contributing to deregulated phenotypes in HTLV-1-infected T cells (Gazon et al., 2017). Tax also interacts with c-Jun NH2-terminal kinases (JNKs), modulating cell proliferation and apoptosis, thereby influencing viral replication and transformation. JNK inhibitors have demonstrated potential in reducing HTLV-1-driven T-cell activation, highlighting the therapeutic significance of this pathway (Bangham and Ratner, 2015).

Tax recruits STAT3 to super-enhancers, increasing chromatin accessibility at loci such as *BCL-xL*, *Myc*, and *IL-21*, driving tumor progression (Jhan and Andrechek, 2016). Super-enhancers play a pivotal role in activating oncogenic gene expression. Meanwhile, HBZ inhibits STAT5, blocking apoptosis and promoting immune escape. It also represses NF-κB activity and innate immune responses (Mohanty and Harhaj, 2023; Enose-Akahata et al., 2017). By suppressing IFN-β production through the inhibition of IRF3 activation, HBZ prevents apoptosis by downregulating pro-apoptotic genes (Chattopadhyay et al., 2011).

Super-enhancers are critical in regulating HTLV-1 transcription. Transcription factors such as CREB/ATF and AP-1 interact with Tax-responsive elements (TREs), driving viral gene expression (Pluta et al., 2020; Giam and Semmes, 2016) These mechanisms enhance cell survival, proliferation, and transformation (Giam and Semmes, 2016; Bellon et al., 2024). Activated STAT3, stimulated by cytokines or growth factors, promotes cell differentiation, proliferation, and resistance to apoptosis, further contributing to malignancy (Wang et al., 2022). Targeting STAT3 with inhibitors like tofacitinib offers a promising therapeutic strategy for suppressing oncogenic pathways driving ATLL (Adesoye et al., 2024).

### Chromatin accessibility and epigenetic reprogramming in HTLV-1 and ATLL

HTLV-1 leverages epigenetic mechanisms to alter host chromatin structure, ensuring persistent infection and promoting oncogenesis (Figure 5). These changes influence gene expression, contributing to the development of ATLL (Mizuike et al., 2025). The virus integrates into transcriptionally active euchromatin regions, often targeting genes associated with cellular growth and survival. This integration facilitates viral persistence and cellular transformation. In infected CD4 + T cells, HTLV-1 replicates through either proviral DNA duplication or transcription into mRNA, enabling the production of viral proteins. The viral transactivator protein Tax plays a critical role in chromatin remodelling, affecting gene expression and RNA splicing (Kim and Lieberman, 2024). HTLV-1 integration frequently occurs near transcription start sites and interacts with key transcription factors like STAT1 and p53, as well as enzymes such as PP2A and BRG1, which regulate transcription at these loci (Ahuja et al., 2014). The viral proteins Tax and HBZ are central to the progression of ATLL by regulating cell signalling and gene expression (Akbarin et al., 2024). Tax activates transcription from methylated HTLV-1 LTRs by interacting with MBD2. Histone modifications associated with transcriptional activation, such as H3K4me3, H3K9ac, and H3K27ac, are frequently observed, while the reprogramming of H3K27me3 by PCR3 disrupts gene regulation and drives ATLL progression. Epigenetic co-activators like EP300 and CBP enhance the expression of genes involved in DNA repair, apoptosis, and proliferation. Concurrently, these epigenetic changes silence tumor suppressor genes, undermining genomic stability (Letafati et al., 2025).

Tax constitutively activates NF-κB, driving chromatin remodelling at inflammatory and survival gene loci. By degrading IκBα, Tax enables NF-κB to regulate genes such as *IL-2*, *IL-9*, and *BCL-xL*, promoting cell proliferation and apoptosis resistance (Bellon et al., 2024). Additionally, Tax recruits BRD4 to sustain NF-κB transcriptional activity, facilitating viral replication and malignancy. This process operates through both canonical pathways, involving phosphorylation of the IKK complex, and non-canonical pathways, mediated by IKKα-dependent processing of p100 to p52 (Su et al., 2024). Targeting these pathways with NF-κB inhibitors shows potential for reversing Tax-mediated malignancies (Hleihel et al., 2023).

HTLV-1 recruits chromatin remodelers such as SWI/SNF, CHD, and INO80 to restructure chromatin. The *Tax* protein interacts with histone-modifying enzymes like HATs and HDACs, recruiting p300/CBP to enhance histone acetylation at H3K9ac and H3K27ac marks, driving transcriptional activation. Conversely, Tax associates with transcriptional repressors like SIRT1 and methyltransferases such as SUV39H1 and SMYD3, influencing transcription through methylation at H3K4 and H3K27 sites (Ratner, 2021; Mizuike et al., 2025). Loss of RUNX3 function also induces chromatin repression through marks like H3K27me3, silencing key apoptosis-related genes (Kulkarni et al., 2018).

Distinct methylation patterns are observed in HTLV-1-infected cells. The 5′ LTR of the proviral genome is hypermethylated, silencing the *Tax* gene, while the 3′ LTR is hypomethylated, sustaining *HBZ* gene expression. These modifications help HTLV-1 evade immune responses and establish latency. Treatments with hypomethylating agents, such as 5-azacytidine, can reactivate viral gene transcription in latently infected cells, emphasizing the regulatory role of DNA methylation in HTLV-1 persistence (Pietropaolo et al., 2021; Mizuike et al., 2025).

Aberrant DNA methylation in ATLL cells affects several host genes critical for cellular function (Geissler et al., 2024). Hypermethylation silences tumor suppressor genes like *CDKN2A* and *BMP6*, leading to unchecked proliferation and altered signalling (Geissler et al., 2024; Wajed et al., 2001). Similarly, genes such as *KLF4* and *EGR3*, crucial for cell cycle regulation and apoptosis, are silenced, enabling resistance to activation-induced cell death (Pietropaolo et al., 2021). Hypermethylation also impacts zinc finger transcription factors and MHC class I proteins, allowing ATLL cells to evade immune detection(Letafati et al., 2025; Paixão et al., 2006). Clinically, the extent of promoter-associated CpG island hypermethylation is correlated with poor prognosis in ATLL patients (Paixão et al., 2006). Preclinical studies suggest that hypomethylating agents could target these epigenetic changes, offering therapeutic potential (Letafati et al., 2025).

## Epstein–BARR virus and cancer

### EBV virology

EBV, also known as human herpesvirus 4 (HHV-4), is a widespread lymphotropic gamma-herpesvirus with a biphasic lifecycle comprising both lytic and latent phases (Cao et al., 2024). This double-stranded linear DNA virus is primarily transmitted through saliva, where it establishes replicative infections in the oropharynx and a lifelong latent presence in oral epithelial cells and B cells (Zhang et al., 2024; Dasari et al., 2023; Parija, 2023). EBV’s persistent infection, often asymptomatic, has oncogenic potential and is associated with several malignancies, including nasopharyngeal carcinoma, EBV-associated gastric carcinoma, Burkitt lymphoma, and Hodgkin lymphoma, particularly in immunosuppressed individuals (Zhou et al., 2025; Looi et al., 2021; Su et al., 2023).

The oncogenic capacity of EBV arises from its large genome, which encodes numerous viral genes capable of manipulating the cellular environment. These genes co-opt PTFs, alter host epigenetic machinery, and influence key oncogenic pathways, producing medically significant viral strains that drive cancer development (Wen et al., 2022; Das and Kundu, 2025).

EBV infects more than 95% of the global adult population, establishing a lifelong latent infection characterized by periodic reactivation and viral shedding (Murata et al., 2021; Damania et al., 2022). The virus demonstrates remarkable adaptability in its gene expression programs, transitioning between lytic replication and multiple latency types. Each latency form is defined by the expression of specific viral genes including Epstein–Barr nuclear antigens (EBNAs, EBNA1-2, 3A-3C) and latent membrane proteins (LMPs), which are crucial for maintaining the viral genome and modulating host immune responses. These programs are tightly regulated by epigenetic modifications and the activity of recruited transcription factors, enabling EBV to adapt to diverse cellular environments while promoting oncogenesis (Kong and Giulino-Roth, 2024).

Epigenetic changes, such as chromatin remodelling and transcriptional reprogramming, play pivotal roles in the development of EBV-associated malignancies. These modifications are key to understanding the mechanisms by which EBV contributes to cancer progression, illustrating how the virus shapes host gene expression to support its lifecycle and oncogenic strategies.

### EBV-mediated regulation of PTFS

**Figure 6:**
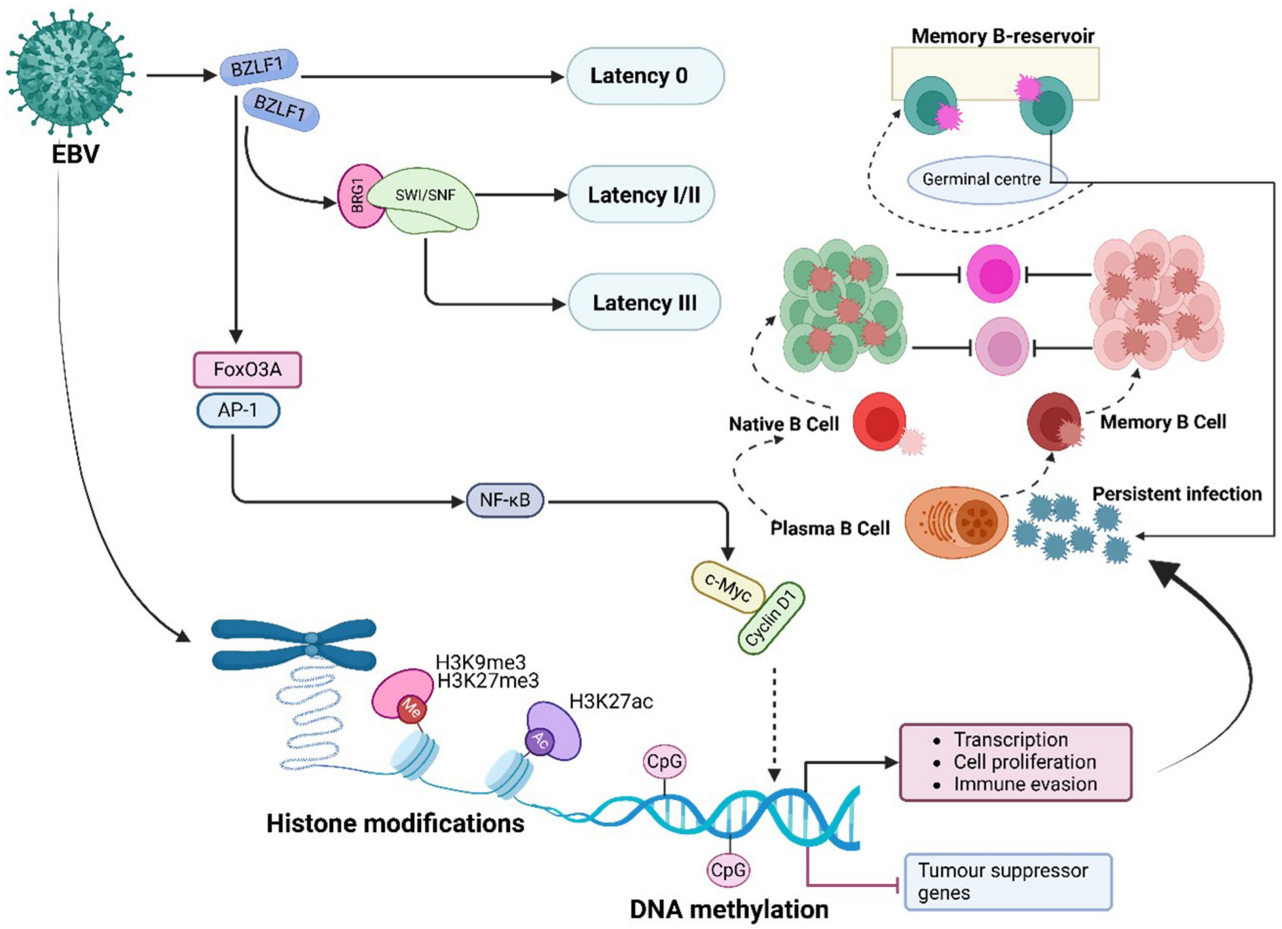
EBV-mediated PTFs regulation, chromatin remodelling and epigenetic reprogramming, and role in oncogenesis. EBV alters host and viral chromatin through histone (e.g., H3K9me3, H3K27me3) and DNA modifications, silencing tumor suppressor genes (e.g., *p16*, *RASSF1A*, *CDH1*, *PTEN*) and activating oncogenic pathways. These changes enhance immune evasion, cell proliferation, and malignant transformation, contributing to cancer development.

EBV manipulates host PTFs to remodel chromatin and sustain oncogenic programs (Figure 6). A central player in this process is the EBV immediate-early protein BZLF1 (also known as Zta or ZEBRA), which functions as a viral pioneer factor facilitating the transition from latency to the lytic cycle (Bernaudat et al., 2022). BZLF1 interacts with chromatin remodelling complexes such as BRG1-containing SWI/SNF and HATs, enabling chromatin opening at viral promoters and initiating lytic reactivation (Asha and Sharma-Walia, 2021). Additionally, BZLF1 counteracts repression mediated by PRC2, activating the transcription of viral genes essential for the lytic phase of infection (Asha and Sharma-Walia, 2021).

Another crucial pTF affected by EBV is FoxO3A, a tumor suppressor that is downregulated in EBV-infected cells. This downregulation disrupts chromatin accessibility at pro-apoptotic gene loci, reducing apoptosis and enhancing B cell survival (Neugebauer et al., 2023; Huang et al., 2021). Loss of FoxO3A function significantly contributes to the development of lymphomas. Similarly, the viral protein LMP1 persistently activates NF-κB signalling in EBV-associated Hodgkin lymphoma, promoting chromatin opening at loci encoding inflammatory cytokines like IL-6 and anti-apoptotic proteins like BCL-2. This activation supports the survival and proliferation of EBV-infected cells (Banerjee et al., 2024; Chakravorty et al., 2022).

In addition to targeting FoxO3A and NF-κB, EBV exploits the AP-1 transcription factor complex (FOS/JUN dimer) to drive oncogenic transcriptional programs (Song et al., 2023). The viral protein LMP1 activates AP-1, which recruits BRD4, a bromodomain protein that amplifies the transcription of oncogenic factors such as *c-Myc* and *CCND1*. In Burkitt lymphoma, EBNA2 works in conjunction with p300, a histone acetyltransferase, to acetylate chromatin at the *c-Myc* locus, enhancing cellular proliferation and contributing to oncogenesis (Kasprzyk et al., 2021).

### Chromatin accessibility in EBV infection and cancer development

EBV undergoes chromatinization upon entering the nucleus (Figure 6). This process involves extensive histone modifications and DNA methylation, which regulate viral gene expression and establish distinct latency programs: Latency I, II, and III (Scott, 2017). Each latency phase is defined by specific chromatin landscapes and is associated with malignancies (Ho et al., 2023). In Latency I, which is typically observed in Burkitt lymphoma, most viral genes are silenced through repressive histone modifications such as H3K9me3 and H3K27me3, alongside DNA methylation (Chen et al., 2024; Shareena and Kumar, 2023). By contrast, Latency II and III, seen in Hodgkin lymphoma and NPC, involve the reactivation of oncogenic viral genes like *LMP1* and *LMP2A* via chromatin-opening mechanisms (Mesri et al., 2014; Zhou et al., 2025). Transitions between these latency programs are tightly regulated by epigenetic factors (Kong and Giulino-Roth, 2024).

Epigenetic modifications are central to EBV’s capacity to manipulate host chromatin, ensuring viral persistence and promoting oncogenic transformation (Dai et al., 2021; Soliman et al., 2021; Zhou et al., 2025). By dynamically altering chromatin accessibility, EBV maintains latency, evades immune responses, drives uncontrolled cellular proliferation, and suppresses tumor suppressor gene transcription (Zhou et al., 2025). For instance, the viral protein LMP1 recruits HATs, such as p300/CBP, to enhance H3K27ac, activating genes involved in cell proliferation and immune evasion (Dai et al., 2021; Soliman et al., 2021; Zhou et al., 2025). Additionally, EBNA3C interacts with histone methyltransferases like EZH2, a component of PRC2, depositing H3K27me3 at tumor suppressor loci. This modification silences genes such as *p16* and *RASSF1A*, contributing to cancer development (Banerjee et al., 2024).

In B cells, EBV infection removes repressive histone marks, including H3K9me3, H3K27me3, and H4K20me3, thereby enhancing chromatin accessibility and regulating genes linked to the cell cycle and apoptosis (Zhou et al., 2025). Viral protein-induced histone modifications destabilize normal cellular processes, facilitating malignant transformation. These changes, affecting both the viral and host genomes, are essential for establishing and maintaining latency while driving cellular transformation (Zhou et al., 2025; Niller et al., 2016).

Both viral and host DNA methylation play crucial roles in the development of EBV-associated cancers. During latent infection, the EBV genome undergoes extensive methylation to regulate viral gene expression. This widespread CpG island methylation, known as the CpG island methylator phenotype (CIMP), is frequently observed in EBV-associated cancers, such as gastric carcinoma and NPC (Salnikov et al., 2024; Leong and Lung, 2021). The viral protein BZLF1 preferentially binds methylated CpG motifs in key viral promoters, facilitating the transition from latency to lytic infection (Zhang et al., 2022). This binding bypasses repressed chromatin states without requiring active DNA demethylation, promoting efficient lytic viral gene expression and ensuring persistence and oncogenic potential (Zhang et al., 2022).

EBV also induces widespread aberrant DNA methylation in the host genome, silencing tumor suppressor genes and advancing tumor progression. Latent membrane proteins (LMP1 and LMP2A) upregulate DNMT1, DNMT3A, and DNMT3B, resulting in global DNA hypermethylation and repression of tumor suppressor genes (Kuss-Duerkop et al., 2018). Key tumor suppressor genes silenced through EBV-induced hypermethylation include *CDH1*, which disrupts cell adhesion and promotes invasion and metastasis, and *RASSF10*, which suppresses apoptosis and cell proliferation (Pietropaolo et al., 2021). Moreover, LMP2A activates the STAT3 pathway to enhance DNMT1 activity, leading to promoter hypermethylation of *PTEN*, thereby driving uncontrolled cell proliferation and survival (Pietropaolo et al., 2021). Hypermethylation of genes such as *CDH1* and *RASSF10* may serve as biomarkers for early detection of EBV-associated cancers. Detecting these methylation changes in tissue biopsies or circulating tumor DNA can aid early diagnosis and improve prognosis assessments.

## Human herpesvirus 8

### HHV-8 virology

Human herpesvirus 8 (HHV-8), also referred to as Kaposi’s sarcoma-associated herpesvirus (KSHV), belongs to the *Rhadinovirus* genus and *Gammaherpesvirinae* subfamily within the herpesvirus family (Gessain, 2008; Sarid et al., 2020). It is a recognized causative agent of Kaposi’s sarcoma (KS), primary effusion lymphoma (PEL), and multicentric Castleman disease (MCD), conditions that are particularly prevalent among immunocompromised individuals, such as those living with HIV/AIDS (Sarid et al., 2020; Chan et al., 2000; Pei et al., 2020). HHV-8 drives cancer development through intricate interactions with the host’s cellular machinery, involving the regulation of transcription factors, chromatin remodelling, and epigenetic reprogramming (Pei et al., 2020).

HHV-8 is predominantly transmitted through saliva, though other routes include sexual contact, blood transfusion, organ transplantation, and, less commonly, mother-to-child transmission (Tan and Pinsky, 2017; Jenkins et al., 2002). The virus infects various cell types, such as endothelial cells, B-cells, monocytes, and epithelial cells, enabling angiogenesis and immune evasion (Knowlton et al., 2013). Individuals with weakened immune systems, including those with HIV/AIDS or transplant recipients, are especially vulnerable to cancers associated with HHV-8 (Sunil et al., 2010).

Structurally, HHV-8 features an enveloped, icosahedral capsid containing a double-stranded DNA genome measuring 140–170 kilobases (Bai et al., 2024). Tegument proteins within the capsid play crucial roles in the virus’s functionality; Its genome is composed of conserved genes responsible for replication, latency, and structural integrity, alongside unique genes that facilitate immune evasion, angiogenesis, and cell proliferation (Bai et al., 2024). Key genes include ORF50 (transcription activator, RTA), which drives reactivation from latency; LANA (ORF73), which aids in latency maintenance and apoptosis inhibition; vIL-6, a cytokine linked to inflammation; and vGPCR, which promotes angiogenesis and cell growth (Losay and Damania, 2025).

The replication cycle of HHV-8 occurs in two phases. During the latent phase, the viral genome exists in the nucleus as circular DNA, with limited gene expression (e.g., LANA, vCyclin) aimed at suppressing apoptosis and evading immune detection (Losay and Damania, 2025). This phase is vital for the virus’s long-term persistence and potential to cause tumours (Losay and Damania, 2025). The lytic phase, triggered by environmental stressors like hypoxia or immune suppression, is characterized by the reactivation of the virus mediated by ORF50 (RTA) (Sarid et al., 2020; Aneja and Yuan, 2017). This leads to the production of new virus particles, cell destruction, and further spread of the infection (Aneja and Yuan, 2017).

### HHV-mediated regulation of PTFS

**Figure 7:**
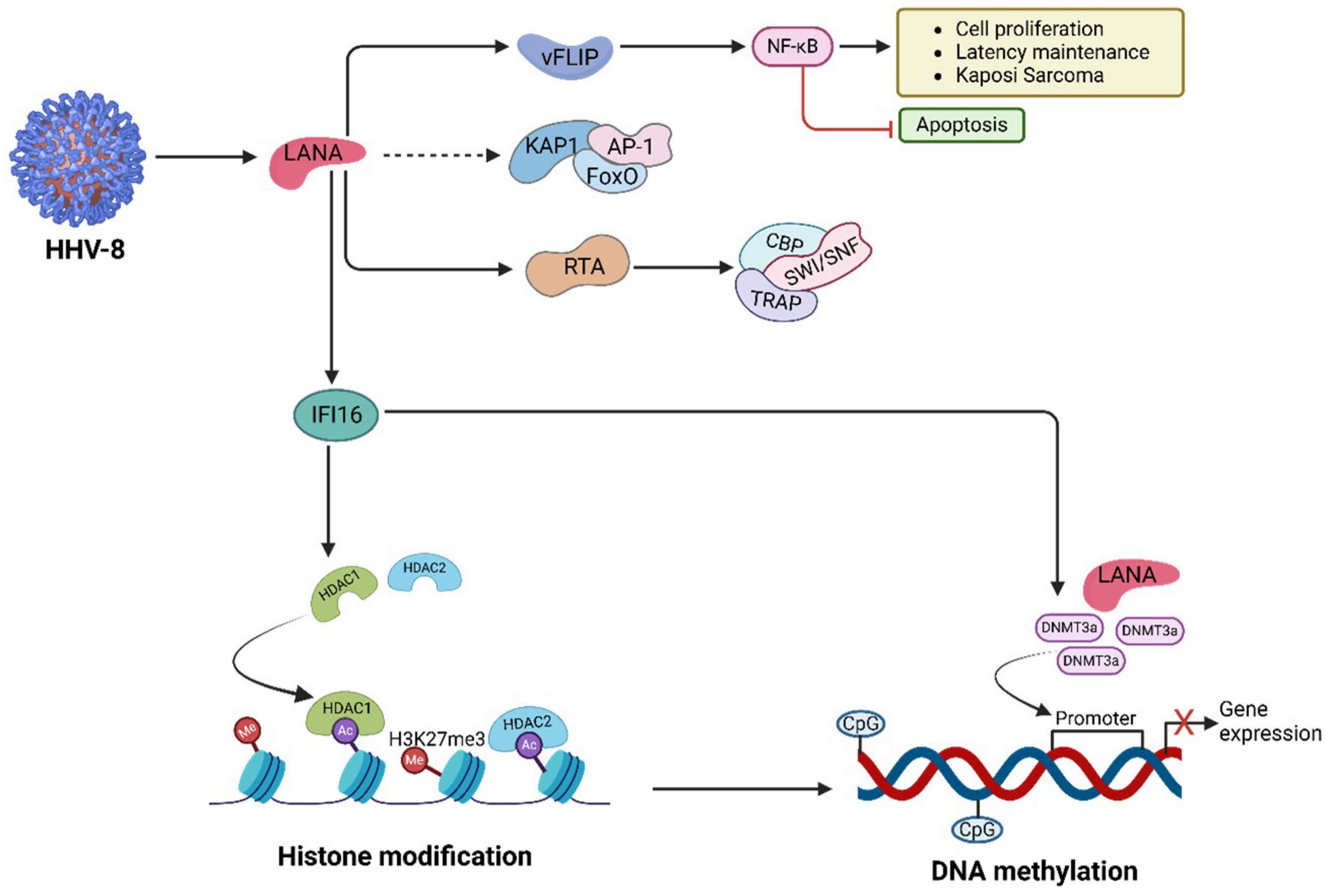
HHV-8-mediated PTFs regulation, chromatin remodelling and epigenetic reprogramming, and role in oncogenesis. HHV-8 proteins such as LANA, vIL-6, and vGPCR, which facilitate immune evasion and angiogenesis. HHV-8 promotes histone modifications and DNA methylation, silencing tumor suppressor genes like *p16INK4a* and *TGF-β receptor*. LANA restructures host chromatin, recruits HDACs and DNMTs, and suppresses apoptosis pathways, enabling viral persistence and oncogenic progression. These processes collectively reshape the chromatin landscape and sustain HHV-8’s oncogenic potential.

HHV-8-induced oncogenesis involves a complex network of molecular pathways that drive cell survival, proliferation, and immune evasion (Figure 7). A central player in this process is the NF-κB signalling pathway, which is activated by viral FLICE inhibitory protein (vFLIP). An et al., (An et al., 2003) demonstrated that vFLIP stimulates NF-κB signalling, leading to increased cell proliferation and suppression of apoptosis. This promotes KS tumorigenesis through unchecked cell growth and resistance to programmed cell death (An et al., 2003). Kang et al., (Kang et al., 2008) further revealed that NF-κB activation supports inflammatory responses and angiogenesis, critical processes for the progression of KS and primary effusion lymphoma (PEL) (Kang et al., 2008).

The replication and transcription activator (RTA) protein of HHV-8 plays a significant role in viral gene expression by interacting with a short acidic sequence in its carboxyl region (Gwack et al., 2003). RTA recruits key factors such as CBP, the SWI/SNF chromatin remodelling complex, and the thyroid hormone receptor-associated protein (TRAP)/Mediator coactivator to viral promoters (Gwack et al., 2003). This recruitment is essential for loosening the nucleosome structure at replication sites, facilitating RTA-driven gene expression. Notably, SWI/SNF-related, matrix-associated, actin-dependent regulator of chromatin subfamily B member 1 (SMARCB1), a tumor suppressor protein involved in hedgehog signalling, is recruited during this process and contributes to regulating tumorigenic pathways (Asha et al., 2020). Additionally, direct interactions between RTA and the Brg1 subunit of SWI/SNF and the TRAP230 subunit of TRAP/Mediator have been identified, emphasizing their involvement in HHV-8 gene regulation (Gwack et al., 2003).

The c-Myc proto-oncogene is another key factor in HHV-8-mediated cancers. Liu et al. (2007) showed that HHV-8’s LANA binds to c-Myc promoter regions, increasing its transcription. This upregulation drives cellular transformation and tumor proliferation in KS, highlighting c-Myc’s pivotal role in HHV-8 oncogenesis (Liu et al., 2007).

HHV-8 also exploits Sp1, a transcription factor, to regulate gene expression supporting cell growth and survival. Verma et al., (Verma et al., 2004) demonstrated that LANA interacts with Sp1 to control genes involved in the cell cycle, leading to the upregulation of pro-survival and proliferative pathways, which further promote KS progression (Verma et al., 2004).

In addition, HHV-8 manipulates AP-1 activity to foster a pro-oncogenic environment by upregulating IL-6 and c-Jun, an AP-1 component, through the MAPK pathway (Yang et al., 2023). This upregulation enhances the transcription of pro-inflammatory and pro-survival genes, sustaining the inflammatory and proliferative conditions characteristic of KS (Yang et al., 2023).

FoxO transcription factors, critical regulators of apoptosis and stress response, are inactivated during HHV-8 infection. Gao et al., (Lan et al., 2023) demonstrated that HHV-8 inhibits FoxO1 activity, suppressing apoptosis and promoting uncontrolled cell proliferation. They also showed that knocking down FoxO1 increases intracellular reactive oxygen species (ROS) levels, which disrupt HHV-8 latency and induce viral lytic reactivation. Similarly, Lan et al. (2023) found that FoxO3 is phosphorylated and inactivated during HHV-8 infection, further enhancing the survival of infected cells and driving tumor progression.

Together, these mechanisms demonstrate how HHV-8 reprograms host signalling and transcriptional machinery to sustain its persistence and promote oncogenesis. By hijacking pathways such as NF-κB, c-Myc, Sp1, AP-1, and FoxO PTFs, HHV-8 creates a favourable environment for cell proliferation, survival, and immune evasion, contributing to the development and progression of KS and other associated cancers.

### Chromatin accessibility and epigenetic reprogramming HHV-8-related KS

HHV-8 drives tumorigenesis by altering histones and chromatin marks, creating conditions that favour viral persistence and oncogenic potential (Figure 7). These modifications involve processes such as histone acetylation, methylation, and deacetylation, which silence tumor suppressor genes while promoting the expression of viral genes. Collectively, these changes support the survival of the virus within host cells and enable cancer development (Han et al., 2024).

LANA is a key protein expressed during HHV-8 latency that plays a central role in maintaining viral latency while restructuring host chromatin. LANA interacts with the host protein IFI16, which facilitates the recruitment of HDAC1 and HDAC2 to specific promoter regions. This recruitment leads to the deacetylation of the RTA promoter, silencing its activity and reinforcing viral latency (Ghosh et al., 2025). By preventing the activation of tumor suppressor pathways, including p53-mediated apoptosis, LANA promotes the survival of infected cells and drives cancer progression (Ghosh et al., 2025). Additionally, LANA recruits the SWI/SNF chromatin remodelling complex to the viral genome, ensuring its stable maintenance within host cells. This mechanism supports viral gene expression during reactivation and underscores the multifaceted role of LANA in HHV-8-mediated oncogenesis (Gwack et al., 2003; Han et al., 2024; Ghosh et al., 2025).

Another host factor manipulated by HHV-8 is KAP1 (KRAB-associated protein 1), a transcriptional co-repressor that contributes to viral latency and oncogenesis (Sun et al., 2014). HHV-8 viral proteins recruit KAP1 to induce chromatin compaction and transcriptional silencing of host genes involved in critical processes such as cell cycle regulation, apoptosis, and immune evasion. These actions enable HHV-8 to establish latency and evade host immune surveillance, significantly promoting cancer progression (Sun et al., 2014).

DNA methylation also plays a pivotal role in HHV-8-mediated tumorigenesis. The viral protein LANA recruits DNMT3a to CpG regions on specific promoters, facilitating DNA methylation and repressing gene expression (Shamay et al., 2006). Additionally, HHV-8 microRNA *miR-K12-4-5p* increases DNMT1, DNMT3a, and DNMT3b levels by targeting their negative regulator, Rbl2, further driving methylation of both viral and host genes (Lu et al., 2010). This alteration silences tumor suppressor genes such as p16INK4a, TGF-*β* type II receptor (TbetaRII/TGFBR2), PDZ-LIM domain-containing protein 2 (PDLIM2), and CDH13, fostering unchecked cellular proliferation characteristic of Kaposi’s sarcoma (Shamay et al., 2006; Sun et al., 2015; Platt et al., 2002; Di Bartolo et al., 2008).

The PRC introduces the H3K27me3 histone mark, further facilitating CpG methylation and contributing to HHV-8 pathogenesis (Schlesinger et al., 2007; Günther and Grundhoff, 2010). Latent viral proteins such as vFLIP and LANA upregulate EZH2, the catalytic subunit of PRC, and assist in recruiting the complex to chromatin, reinforcing the oncogenic potential of the virus (Schlesinger et al., 2007; Günther and Grundhoff, 2010; He et al., 2012; Toth et al., 2016).

Together, these findings emphasize HHV-8’s ability to manipulate host epigenetic and chromatin regulatory mechanisms, including histone acetylation, methylation, and DNA methylation. By hijacking these pathways, the virus silences tumor suppressor genes, reshapes the chromatin landscape, and sustains its oncogenic potential. This highlights the intricate strategies employed by HHV-8 to drive tumorigenesis and persist within host cells.

## Merkel cell polyomavirus

### MCV virology

Merkel cell polyomavirus (MCV) is a human virus that plays a central role in the development of Merkel cell carcinoma (MCC), a rare and aggressive neuroendocrine skin cancer (Houben et al., 2023). MCV is present in over 80% of MCC cases, and its oncogenesis is linked to the viral integration into the host genome and the persistent expression of viral proteins (Feng et al., 2008; Pedersen et al., 2024).

MCV is a non-enveloped virus with a 45–50 nm icosahedral capsid composed primarily of the major structural protein VP1, which enables binding to host cell receptors (Houben et al., 2023; Feng et al., 2008; Pedersen et al., 2024). Minor capsid proteins VP2 and VP3 assist in capsid assembly and facilitate viral entry and genome release during infection (Houben et al., 2023).

MCV’s circular, double-stranded DNA genome is about 5,400 base pairs long and divided into early and late regions (Houben et al., 2023). The early region encodes regulatory proteins, including Large T antigen (LT) and Small T antigen (sT), which drive viral replication and manipulate host cell machinery. LT is critical for initiating viral DNA replication and inactivating tumor suppressor proteins like p53 and pRB, enabling cell survival and proliferation (Houben et al., 2023). Meanwhile, sT stabilizes LT, disrupts cell cycle regulation, and controls viral replication and oncoprotein expression by targeting the cellular ubiquitin ligase SCFFbw7 (Kwun et al., 2013). The late region encodes structural proteins (VP1, VP2, VP3) essential for forming new virus particles (Houben et al., 2023).

Key regulatory elements in the MCV genome include the origin of replication (Ori), promoters, enhancers, and a polyadenylation signal, are critical for MCV clonal integration in the host (Feng et al., 2008; Tolstov et al., 2009; Stakaitytė et al., 2014). These elements ensure precise viral DNA replication and gene expression, enabling the successful production of viral proteins and replication of the virus (Houben et al., 2023; Tolstov et al., 2009). The oncogenic process driven by MCV involves a complex interplay between pioneering transcription factors, chromatin remodelling, and epigenetic reprogramming, which collectively influence the cellular environment to promote malignant transformation.

### MCV-mediated regulation of PTFS

**Figure 8:**
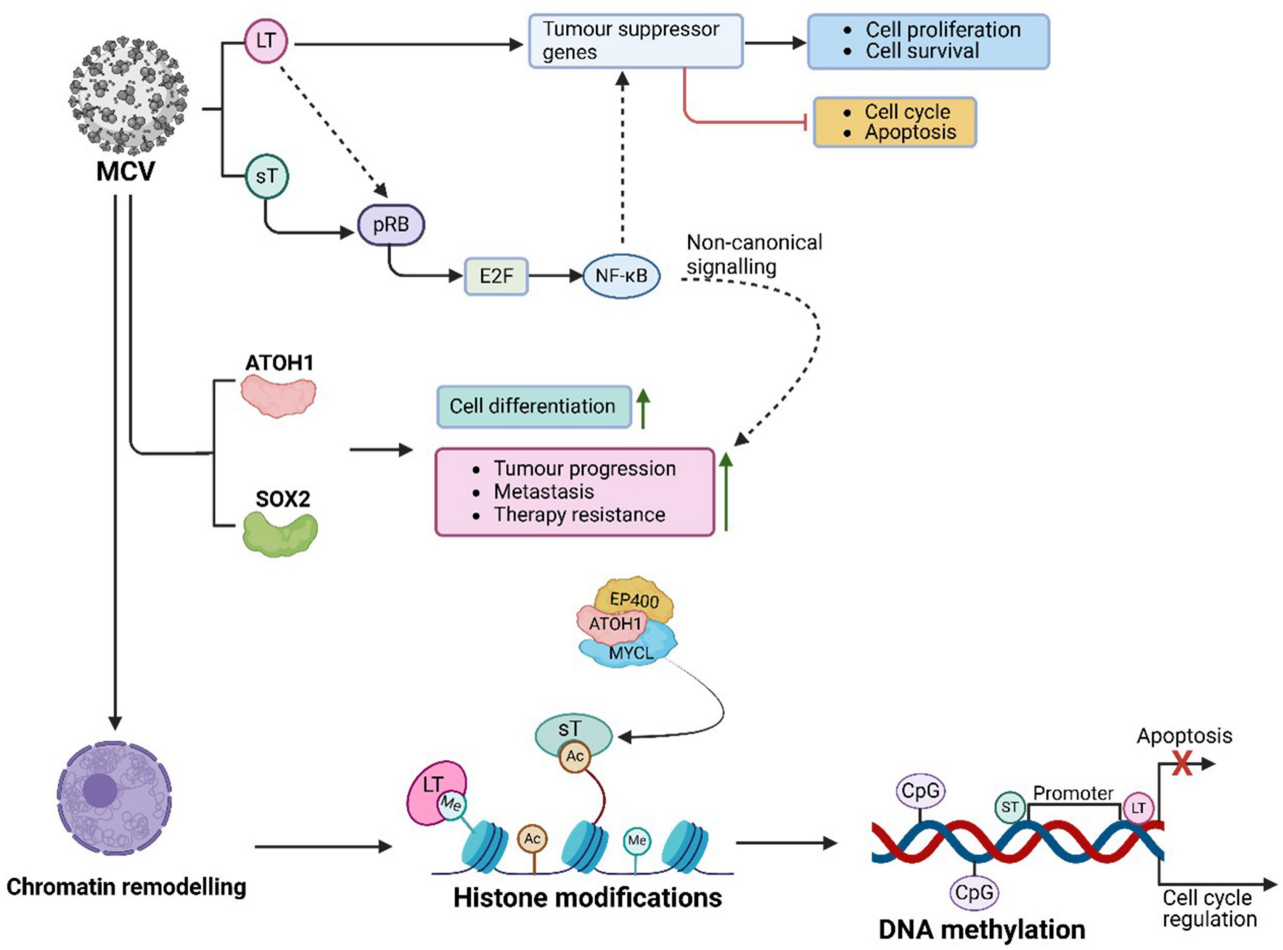
MCV-mediated PTFs regulation, chromatin remodelling and epigenetic reprogramming, and role in oncogenesis. MCV LT inactivates tumor suppressors (e.g., *p53, pRB*), recruits chromatin modifiers, and releases E2F transcription factors to promote cell cycle progression. Meanwhile, sT regulates complexes like MYCL and EP400, contributing to chromosomal instability and oncogene activation. MCV induces epigenetic reprogramming, including chromatin remodelling, histone modifications, and methylation of genes like *CDKN2A* and *RASSF1A*, silencing tumor suppressor genes and enhancing immune evasion. This reshaping of the chromatin landscape activates oncogenes, disrupts cellular processes, and drives MCC progression.

MCV LT plays a central role in oncogenesis by targeting and inactivating the tumor suppressor proteins p53 and pRB (Figure 8). This disruption prevents cell cycle arrest and apoptosis, enabling uncontrolled cellular proliferation and survival. Shuda et al., (Shuda et al., 2008) demonstrated that LT binds to and inactivates these tumor suppressor pathways, allowing cells to bypass critical checkpoints and divide uncontrollably, a hallmark of tumorigenesis (Shuda et al., 2008). Moreover, LT, in conjunction with the ST antigen, further disrupts cellular checkpoints by targeting both *pRB* and *p53* genes, leading to chromosomal instability and mutation accumulation, which drive MCC progression (Pedersen et al., 2024; Kellogg et al., 2022; Helmbold et al., 2009).

The inactivation of pRB1 by LT also results in the release of E2F transcription factors, which are crucial regulators of the cell cycle (Pedersen et al., 2024; Hesbacher et al., 2016). These E2F transcription factors are upregulated in MCV-positive MCC cells following pRB1 inactivation by LT. This upregulation drives the transcription of genes necessary for DNA replication and cell division, facilitating unchecked proliferation and contributing to the oncogenic phenotype of MCC (Pedersen et al., 2024; Hesbacher et al., 2016).

Atonal Homolog 1 (ATOH1) and SOX2 are PTFs that play vital roles in Merkel cell differentiation, and tumor-suppressing pathways (Harold et al., 2019). ATOH1, a basic helix–loop–helix (bHLH) transcription factor, is essential for the differentiation of neuroendocrine cells such as Merkel cells, which are involved in touch perception. Its expression in MCC correlates with advanced disease progression, underscoring its prognostic significance (Gambichler et al., 2017). When ATOH1 function is disrupted, tumor cells may lose their Merkel cell identity and acquire stem-like characteristics, leading to malignancy.

SOX2, known for its role in maintaining stem cell properties and pluripotency, is another key factor in sustaining the MCC phenotype. Frequently upregulated in MCC, particularly in MCV-positive tumours, SOX2 fosters a stem-like state that promotes tumor progression, metastasis, and therapy resistance (Harold et al., 2019). MCV LT drives the expression of both SOX2 and ATOH1 by inhibiting pRB, thereby maintaining the MCC phenotype. Notably, knocking down LT in MCV-positive MCC cells results in the loss of SOX2 and ATOH1 expression and induces a phenotypic conversion into a differentiated neuronal state. This transformation can also be triggered by inhibiting SOX2 alone, highlighting its pivotal role in maintaining MCC characteristics (Harold et al., 2019).

The viral oncoproteins, including LT and sT, also activate the non-canonical NF-κB signalling pathway, which supports MCC progression (Zhao et al., 2020; Gerer et al., 2017). NF-κB promotes tumor growth by driving the transcription of genes that inhibit apoptosis, advance the cell cycle, and enable immune evasion. Additionally, it contributes to inflammation, a key feature of the tumor microenvironment, and enhances the expression of pro-survival proteins such as BCL-2 and inhibitors of apoptosis (IAPs), thereby fostering MCC cell survival, proliferation, and therapeutic resistance (Zhao et al., 2020; Gerer et al., 2017).

In MCV-related MCC, the YAP/TEAD signalling axis influences gene expression related to cell proliferation, survival, and stem-like characteristics (Frost et al., 2023). Notably, there is an inverse correlation between neuroendocrine gene expression and the activity of YAP1 and WW domain-containing transcriptional regulator 1 (WWTR1) at both the transcript and protein levels in MCC samples (Frost et al., 2023). In MCV-positive MCC, YAP1 and WWTR1 induce cell cycle arrest by repressing LT expression through TEAD-dependent transcriptional mechanisms. This underscores the importance of YAP1/WWTR1 silencing in the development of MCV-related MCC and highlights the heterogeneity of neuroendocrine gene expression in MCC (Frost et al., 2023). Acting as PTFs, the YAP/TEAD complex can influence chromatin remodelling at critical regions involved in cell growth and survival, further establishing its role in MCC progression.

### Chromatin accessibility and epigenetic reprogramming in MCV-related MCC

The integration of MCV into the host genome is a defining feature of MCC (Shuda et al., 2008). This integration often results in the persistent expression of viral proteins, including LT and ST antigens, which significantly alter chromatin structure and regulate gene expression (Rotondo et al., 2021). Feng et al. (2008) demonstrated that MCV integration disrupts tumor suppressor genes such as *p53* and *pRB*, triggering chromatin rearrangements that activate oncogenes and promote cellular transformation (Feng et al., 2008). LT further contributes to tumorigenesis by binding to host chromatin, remodelling it to upregulate pro-proliferative genes (Feng et al., 2008; Lu et al., 2012). Sheng et al. (2018) also found that MCV integration modifies host chromatin methylation patterns, activating genes involved in cell cycle progression and suppressing apoptosis, ultimately facilitating both viral replication and oncogenesis.

Histone modifications play a pivotal role in regulating gene expression in MCV-associated MCC (Park et al., 2020; Sheng et al., 2018; Cheng et al., 2017). LT and ST drive histone modifications at key promoter regions to suppress apoptosis-related genes and activate those essential for cell cycle progression (Park et al., 2020; Sheng et al., 2018; Cheng et al., 2017). These viral proteins also interact with chromatin-modifying enzymes, with LT recruiting HDACs and chromatin remodelers to suppress tumor suppressor genes while activating genes that promote cell survival and proliferation (Sheng et al., 2018).

ATOH1, plays a crucial role in chromatin remodelling, regulating cell cycle progression, differentiation, and apoptosis (Park et al., 2020; Cheng et al., 2017). In MCC, ATOH1 promotes oncogenesis by recruiting MYCL to the EP400 histone acetyltransferase and chromatin remodelling complex (Cheng et al., 2017). However, Park et al., (Park et al., 2020) demonstrated that ST represses ATOH1 expression by forming a complex with MYCL and EP400 to activate lysine-specific demethylase 1A (LSD1A), REST corepressor 2 (RCOR2), and insulinoma-associated protein 1 (INSM1). LSD1 inhibition reduces MCC growth, emphasizing the antagonistic relationship between LSD1 and the non-canonical BAF chromatin remodelling complex in gene regulation and tumorigenesis (Park et al., 2020; Sheng et al., 2018; DeCaprio, 2021). Similarly, SOX2 aids chromatin remodelling, regulating cancer progression genes and preserving cancer stem cell characteristics, further solidifying the combined role of ATOH1 and SOX2 in MCC oncogenesis (Harold et al., 2019; DeCaprio, 2021).

MCV MT recruits Src family kinases via its SH3 motif, leading to its phosphorylation at the SH2 domain (Peng et al., 2023). Phosphorylated MT then recruits and activates phospholipase C gamma 1 (PLC*γ*1), which triggers inflammatory signalling via NF-κB. NF-κB activity promotes chromatin remodelling and gene expression changes, further linking these processes to histone modifications and tumorigenesis (Peng et al., 2023).

DNA methylation also plays a central role in MCV-driven oncogenesis. This epigenetic reprogramming silences tumor suppressor genes, contributing to tumor progression. Sato et al., (Helmbold et al., 2009) observed the frequent silencing of genes such as *CDKN2A* (encoding p16INK4a) and *RASSF1A* in MCV-positive MCC tumours, facilitating immune evasion and unchecked proliferation (Helmbold et al., 2009). LT and sT mediate this silencing by reshaping the chromatin landscape to favour tumorigenesis.

Additionally, PCR2-mediated reduction of the suppressive H3K27me3 histone mark has been observed in MCV-positive MCC tumours, suggesting its involvement in MCC pathogenesis (Busam et al., 2017). Wang et al., (Wang et al., 2012) further demonstrated that LT and sT interact with bromodomain protein 4 (Brd4), reshaping the host chromatin landscape to drive uncontrolled cell growth. Brd4, which regulates transcriptional activity by binding to acetylated lysines on histones, interacts with LT and colocalizes with the viral replication origin complex in the nucleus, enabling viral DNA replication by recruiting replication factor C (RFC) (Wang et al., 2012). Disrupting the Brd4-LT interaction halts replication, while blocking Brd4’s chromatin role enhances viral replication, revealing distinct functions in viral replication and transcription regulation (Wang et al., 2012).

Together, these studies reveal how MCV manipulates chromatin and epigenetic machinery, including histone acetylation and methylation, to integrate into the host genome, alter gene expression, and drive MCC progression. This hijacking of the host’s regulatory pathways highlights MCV’s oncogenic potential.

### Therapeutic implications

Oncogenic viruses such as HBV, HCV, HPV, HTLV-1, HHV-8, and MCV significantly impact cancer progression by altering epigenetic programming and chromatin accessibility, leading to disrupted gene expression and tumorigenesis. Addressing these challenges, innovative therapies target epigenetic mechanisms and transcriptional dysregulation to counteract viral effects as described in Table 1.

For HBV and HCV, DNMT inhibitors (e.g., decitabine) and HDAC inhibitors (e.g., vorinostat) reverse epigenetic silencing of tumor suppressor genes, while BET inhibitors and CRISPR technologies provide new avenues for managing viral integration and oncogenesis (Zhang et al., 2019; Wu et al., 2020). When combined with direct-acting antivirals (DAAs), HDAC and DNMT inhibitors aim to reduce the long-term risk of HCC associated with HCV infection (Dai et al., 2024). HPV-induced cancers are also managed with HDAC and DNMT inhibitors, YAP/TEAD pathway inhibitors, and immunotherapies like pembrolizumab, offering comprehensive treatment options (Bertagnin et al., 2023; Martinez et al., 2022; Martinez et al., 2022; Yao et al., 2024; Zeidan et al., 2022; Jabbour et al., 2017; Pan et al., 2023; Yang et al., 2021).

Epigenetic therapies also target EBV-driven malignancies, leveraging DNMT and HDAC inhibitors to reverse viral-induced changes, while BET inhibitors and immune checkpoint therapies enhance the host immune response (Leong and Lung, 2021; Sarkozy et al., 2020; Julia and Salles, 2021; Nastoupil et al., 2023). Similarly, in HTLV-1-associated cancers, DNMT and HDAC inhibitors, BET inhibitors, and CRISPR-based techniques mitigate oncogenic processes by disrupting viral transcription and chromatin modifications (Letafati et al., 2025; Wang et al., 2024; Sarkozy et al., 2020; Julia and Salles, 2021; Nastoupil et al., 2023; Li et al., 2018; Smith et al., 2016). BET inhibitors, which target BRD4, inhibit the progression of Tax-infected cells, while emerging CRISPR technologies show potential for targeting key oncogenic genes, such as Tax and HBZ, to mitigate the virus’s impact (Letafati et al., 2025; Wang et al., 2024; Li et al., 2018; Smith et al., 2016).

For HHV-8-associated malignancies, therapeutic strategies include inhibiting NF-κB signalling, reversing viral latency through HDAC, DNMT, and EZH2 inhibitors, and suppressing IL-6/AP-1 signalling (Okpara et al., 2024; Naimo et al., 2021; Hopcraft et al., 2018; Li et al., 2019; Lu et al., 2023; Murphy et al., 2022). Chromatin remodelling inhibitors, FoxO activators, and immune therapies enhance tumor suppression, while antiviral agents like ganciclovir and RTA activators target viral replication and latency.

MCV-related MCC therapies focus on restoring tumor suppressors (e.g., *p53*, *pRB*), targeting transcription factors (ATOH1, SOX2), and modulating pathways like NF-κB and YAP/TEAD. Additionally, epigenetic interventions and checkpoint inhibitors offer a multifaceted approach to managing MCC (Harms et al., 2022; Stewart et al., 2009; Rizzitano et al., 2016; Dombret et al., 2014; Ocana et al., 2015).

These diverse, virus-specific approaches highlight the advancement of therapeutic options targeting the intricate molecular mechanisms underlying oncogenic viruses, offering promising directions for combating virus-associated cancers.

## Conclusion and future research perspectives

Oncogenic viruses, including HBV, HCV, HPV, HTLV-1, EBV, HHV-8, and MCV, employ sophisticated strategies to manipulate host chromatin architecture and epigenetic mechanisms, driving persistent infection and tumorigenesis. These viruses exploit pathways to silence tumor suppressor genes, promote uncontrolled proliferation, and evade immune responses. For example, HBV and HCV reprogram chromatin and epigenetic landscapes to sustain infection and foster the inflammatory microenvironment that leads to hepatocellular carcinoma. HPV utilizes chromatin remodelling and transcriptional reprogramming to dysregulate key pathways like YAP-mediated signalling, silencing tumor suppressor genes through DNA hypermethylation and histone modifications. Similarly, EBV and HTLV-1 alter host gene expression through viral proteins interacting with chromatin remodelling complexes, while HHV-8 activates NF-κB signalling and disrupts transcription factor activity to sustain its oncogenic state. MCV impacts chromatin structure through epigenetic enzyme recruitment and transcription factor regulation, promoting proliferation, chromosomal instability, and immune evasion in MCC. Collectively, the ability of these viruses to hijack host regulatory processes underscores their oncogenic potential and highlights critical areas for therapeutic intervention.

Future research on oncogenic viruses should prioritize exploring how these pathogens manipulate epigenetic mechanisms, including histone modifications, DNA methylation, and chromatin remodelling complexes, to drive tumorigenesis. This knowledge could inform the development of epigenetic therapies, such as HDAC and DNMT inhibitors, tailored to virus-driven malignancies. Additionally, detailed investigations into the interactions between viral proteins and host transcription factors, such as YAP, c-Myc, SOX2, and NF-κB, may reveal novel therapeutic targets to disrupt the oncogenic reprogramming of transcriptional networks. Understanding immune evasion mechanisms employed by these viruses to bypass immune responses is another vital area of study, with potential to enhance immune checkpoint inhibitor efficacy when combined with epigenetic therapies. The identification of virus-specific biomarkers reflecting chromatin and epigenetic alterations could enable earlier detection and personalized treatment strategies for virus-associated cancers. Furthermore, research into resistance mechanisms against therapies, including checkpoint inhibitors and epigenetic drugs, could guide the development of more durable treatment options. Lastly, a deeper examination of the interplay between chromatin remodelling complexes and viral oncoproteins could uncover innovative approaches for interrupting the progression of virus-driven malignancies. These perspectives offer promising opportunities for advancing the understanding of oncogenic viruses and designing effective, targeted interventions.
